# FE vibration analyses of novel conforming meta-structures and standard lattices for simple bricks and a topology-optimized aerodynamic bracket

**DOI:** 10.1038/s41598-020-78239-9

**Published:** 2020-12-08

**Authors:** Todd Doehring, William Nelson, Thomas Harris, Alan Freed

**Affiliations:** 1 ABĒMIS LLC Research Laboratory, Cleveland, OH 44118 USA; 2 J. Mike Walker ‘66 Department of Mechanical Engineering, Texas A&M University, College Station, TX 77843-3123 USA; 3 US Army Research Laboratory, Aberdeen Proving Ground, MD USA

**Keywords:** Materials science, Mathematics and computing, Engineering, Aerospace engineering, Biomedical engineering, Civil engineering, Mechanical engineering

## Abstract

Additive manufacturing (AM) enables production of components that are not possible to make using traditional methods. In particular, lattice-type structures are of recent interest due to their potential for high strength-to-weight ratios and other desirable properties. However, standard periodic lattice structures have problems conforming to complex curved and multi-connected shapes (e.g. holes or sharp-to-smooth mating edges). In addition, standard lattices have well known shear and fatigue weaknesses due to their periodic basis/structure. To address these problems, we developed a new type of shape-conforming meta-structure (HGon) that extends lattices, enabling automated conforming to complex shapes and parametric meta-topology control. HGons also have unique vibration dampening and optimization capabilities. This study presents initial FE analyses of (Part 1) dynamic vibration responses of new HGon meta-structures compared with periodic lattices of equivalent density for a series of basic rectangular structures and (Part 2) a complex multi-connected aerodynamic bracket with field-based stress meta-topology optimization. Results show significantly enhanced vibration dampening behavior and superior strength-to-weight ratios for HGon meta-structures as compared to standard lattices.

## Introduction

New additive manufacturing (AM) technologies enable production of complex structures and components that have not been possible to make using standard manufacturing methods. In particular, 3D lattice structures have shown potential for improved strength-to-weight ratios^1^, light-weighting, and other desirable performance characteristics^2–6^.

**Figure 1:**
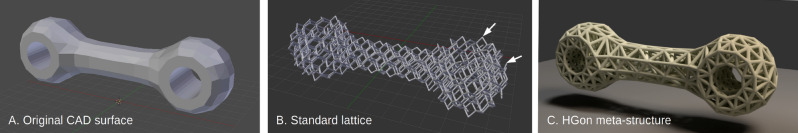
(**A**) CAD surface model of a linkage, (**B**) standard BCC lattice, and (**C**) a new HGon meta-structure. The standard periodic lattice shows common problems of multiple ‘hanging’ struts and edges (arrows) which require manual correction. In contrast, the HGon meta-structures automatically conform precisely and accurately to the original CAD surfaces, edges, and holes with little or no correction required.

Recent reviews^7,8^ have detailed the many advantages (and limitations) of lattices for a wide range of applications. However, for complex shapes (with holes) or highly curved surfaces, periodic lattices often result in ‘hanging’ edges and walls^9^ requiring tedious manual correction procedures (Fig. 1). Lattices also have well known problems of shear/bending weakness and buckling (i.e. ‘house-of-cards’ issues)^3^. Voronoi lattice structures can better fit to curved surface topologies and are useful for design of compliant foam-like structures—e.g. for bio-interfacing applications^11^, however they tend be much weaker under load^10^ and have limited (or no) ability to capture sharp edges and precise boundaries for directed supports, mating/contacting surfaces, or needs such as edges and flat surfaces for CNC and other post-machining requirements.

A number of studies have reported density-graded and ‘conforming’ lattices with cell-periodic structure; warped or otherwise deformed to more complex object boundaries. Han et al.^11^, described a method for uni-axial grading of thickness of AM printed lattices with micro-topology surface characterization. Yuan et al.^12^, developed a cell-based approach to generate lattice-like ‘auxetic’ repeating structures with varying densities. Robbins et al.^13^ developed a warping method to deform cell-based structures to more complex shapes using a topology optimization procedure. Several similar techniques using warping or deformed lattices have been^14–17^ and are currently being used in commercial software products; e.g. nTopology [ntopology.com], Siemens NX^18^, and Autodesk Netfabb^19^. However, an ongoing issue is that all of these methods are based on *repeating cells*, which have fundamental difficulty fitting/conforming to more complex shapes with holes, sharp edges, and complex surface curvatures.

**Figure 2:**
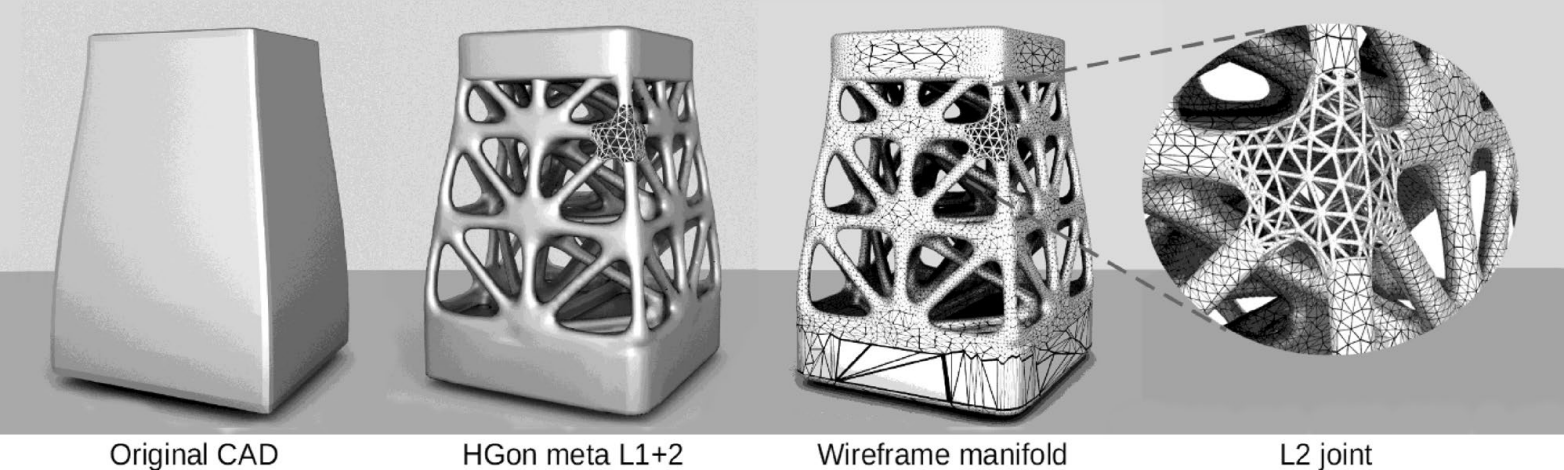
A simple example HGon Level 1 + 2 meta-structure computed from a CAD model (vibration damping platform support). The closeup (L2 joint) further illustrates the meta-structure concept. By varying L1 and L2 densities and topologies the overall structure can be ‘tuned’ to damp desired vibration spectra. Meta-structure generation and volumetrics are programmed in Python (http://python.org) and CuPy (http://cupy.dev) with 3D visualization using Blender3D (http://blender.org).

**Figure 3:**
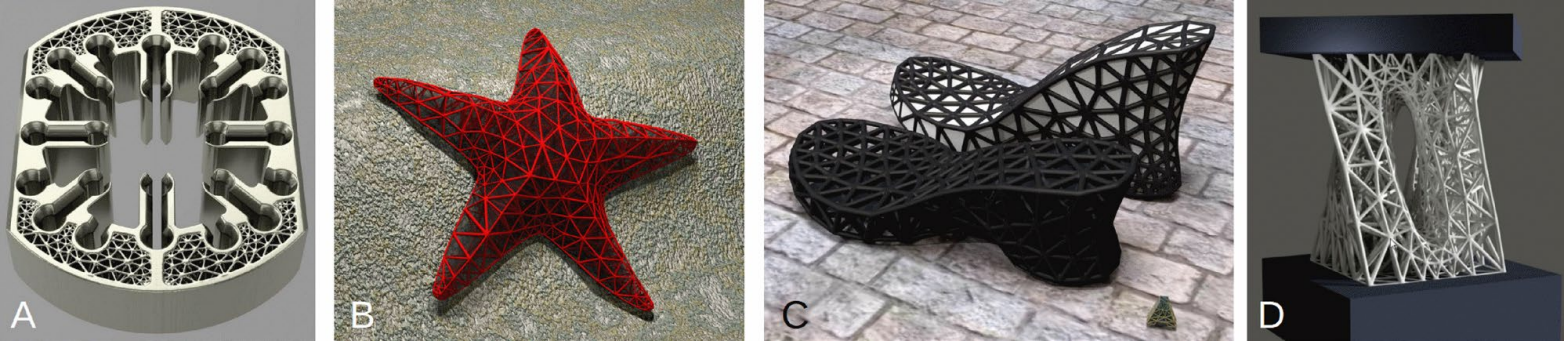
Examples of HGons applied to complex topologies: (**A**) a flow-optimized nuclear fuel rod cassette, (**B**) a ‘starfish’ biomimetic structure, (**C**) footwear Hgons, and (**D**) a multiply-connected HGon under high load-deformation having both sharp linear and smooth curved features.

**Figure 4:**
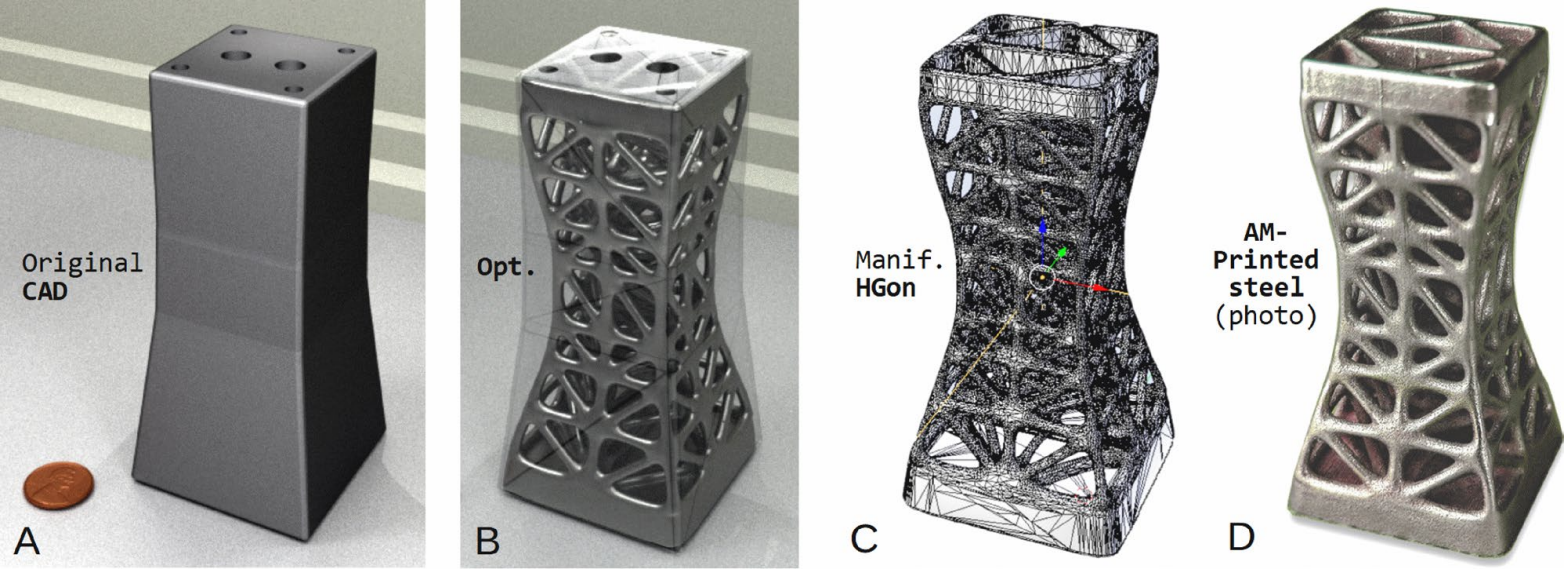
(**A**) CAD bracket beam support, (**B**) tension-shear optimized meta-structure, (**C**) fully manifold HGon polygon STL-surface ready to print, and (**D**) photo of the AM-3D printed component in sintered steel, ready for post-machining.

**Figure 5:**
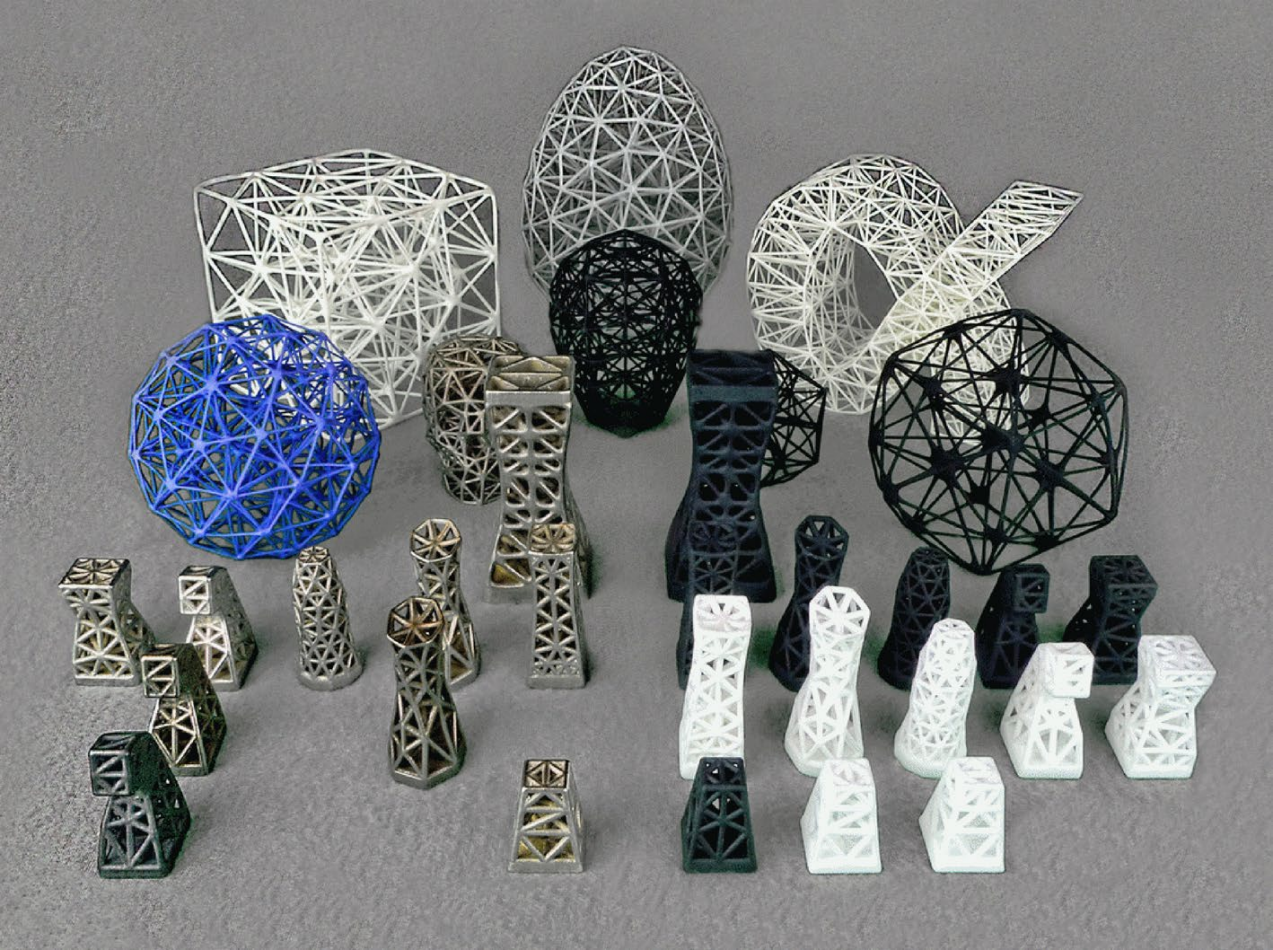
A menagerie of AM-printed HGon structures with wide-ranging simple and complex shapes. Several materials and processes are shown including SLS (sintered) alloys of steel, bronze, brass, aluminum, titanium and plastics e.g. nylon and ABS, Jet-fusion, and SLA printed resin. See the “Appendix” for further information and 3D-VR views of these and other meta-structures.

To address these and related issues, we describe a new type of conforming meta-structure that we call hyper-structures. Hyper-structures (HGons) are a meta-structural generalization of lattices, enabling iterative meta-mesh networks with both periodic and non-periodic (isotropic) meta-topologies, achieving conformity to highly curved and multi-connected shapes (holes, etc.). A novel mathematical representation, automated generation algorithms and custom FE meta-mesh generation methods have been developed for wide ranging shapes and structures (Figs. 2, 3, 4, 5). HGons also have unique capabilities for controlling joint and strut shape, size, taper, chamfer, and other meta-topology parameters as described in the methods and recent utility patent (#10585420)^20^.

HGon meta-structures have been computed and physically produced (3D printed) using latest AM systems and many materials including sintered metals (laser, electron beam), plastics, and multi-material composites. Because HGon meta-structures have aperiodic regions (non-cellular) they can uniquely conform to highly curved shapes, multi-connected solids/surfaces, and both sharp and smooth edges as shown in Figs. 3, 4, and 5.

An interesting way to envision HGon meta-structures are as 3D ‘shadows’ of a higher dimensional parametric shape. In the same sense that a ‘profile’ is a 2D projection of a 3D object; an HGon is a 3D projection of an object defined in 4D, 5D, or higher space. HGon projections (hD-to-3D) can be computed from any combination of structural nodes (points), edge networks (local or global), 3D hulls/primitives, or most any other explicit-implicit topologies. For example, in Part 1 of this study the struts for the simple rectangular shapes were held constant (nominal 2.4 mm dia.) to enable direct comparison. In Part 2, strut-joint radii and local–global density were varied (optimized) by mapping computed fields to local meta-parameters such as strut length, surface-fit criteria, signed distance from boundary, von Mises stress field, and strain energy field. Meta-parameters can be assigned locally and globally such as joint thickness, chamfer, strut taper, and connecting surfaces (e.g. for CNC machining or other post processing).

Hgon meta-structures also have interesting potential for enhanced *vibration dampening* and frequency response control. Because HGons have both isotropic and directed structures, stresses and vibrations are distributed in unique ways. Qualitatively, when one holds an HGon in the hands, it immediately feels ‘strange’. Some reactions are: “It feels like it’s not there…” and “This is metal, but it feels like stone…”.

A primary goal of this initial study was to analyze this unique HGon vibration behavior using dynamic FE methods; directly comparing HGon dynamic response with equivalent periodic lattice and shell structures. Several studies have investigated vibration behavior of simple lattice-like structures, showing increased damping^2^ and resonance suppression capabilities^21^. However, to our knowledge this is the first reported analyses of *load-release* dynamic vibration behavior of periodic lattices, with equivalent cuboid meta-structures and more complex Hgon-type meta-structures.

HGons can now be physically produced (3D printed, CNC finished) to robust industry standards with newly available AM technologies such as the EXOne + SLS metal (exone.com) and VELO3D Sapphire 3-D metal systems (velo3D.com). A variety of HGon structures with engineering-class materials; including metals, plastics, ceramics, bio-compatible and composites with wide ranging complex topologies and sizes have been successfully printed (examples, Fig. 5).

This *2-part* study compares vibrations of solid/shells, standard periodic face-centered-cubic (FCC) lattices, and newly developed HGon meta-structures. In *Part 1*, a simple rectangular cuboid (50 × 50 × 100 mm) solid is used as a basis for comparing vibration responses of varying density lattices and equivalent HGon meta-structures. In *Part 2*, vibration analyses of a complex aerodynamic bracket uses von Mises stress based meta-topology optimization to improve HGon meta-structure and z-axis load carrying performance (up to 72% mass reduction, 5× stiffness).

Our primary *hypotheses* were: (H1) high frequency vibrations would be reduced for HGon meta-structures versus solid/shell and periodic lattices, and (H2) overall dampening would be higher for HGon meta-structures compared with the solid/shell and lattices. Secondary hypotheses were that peak stresses would be reduced for HGons compared to lattices and further after a stress-optimization procedure.

A significant challenge was developing a robust method capable of *automatically* generating FE meta-meshes for these complex HGon meta-structures. The methods and analyses developed and described here can now be applied to most any complex multi-connected, multi-domain structure or shape from 3D-CAD or 3D-hull topologies (e.g. STL, PLY, STEP).

## Methods

### HGon meta-structure generation

Meta-structures were generated by a unique process that combines both explicit and implicit descriptions. The basic meta-structure generation procedure starts by (**step 1**) generating the initial 3D mesh structure from the shape of interest, usually from a triangular STL surface/solid or similar format. Both Delaunay and fractal refinement^22^ are used for the initial ‘base’ meta-mesh, with added steps for HGon-type and hybrid tetrahedral-hexahedral structures. Next, (**step 2**) structural primitives (cylinders, spheres, and/or beams) are accurately positioned at the nodes and edges to generate an initial meta-shape. Then, (**step 3**) for the same nodes and edges, *locally implicit meta-structures* are added (similar to alpha shapes^23^ capsules, spheres, etc.) with accurate positioning as per step 2. These implicit structures add novel local parametric shape control that cannot be done using explicit structures alone. Finally, (**step 4**) the explicit and implicit local meta-structures are combined using a proprietary algorithm that automatically extracts an optimized 3-D *manifold* meta-shape (HGon) based on the input meta-parameters.

**Figure 6:**
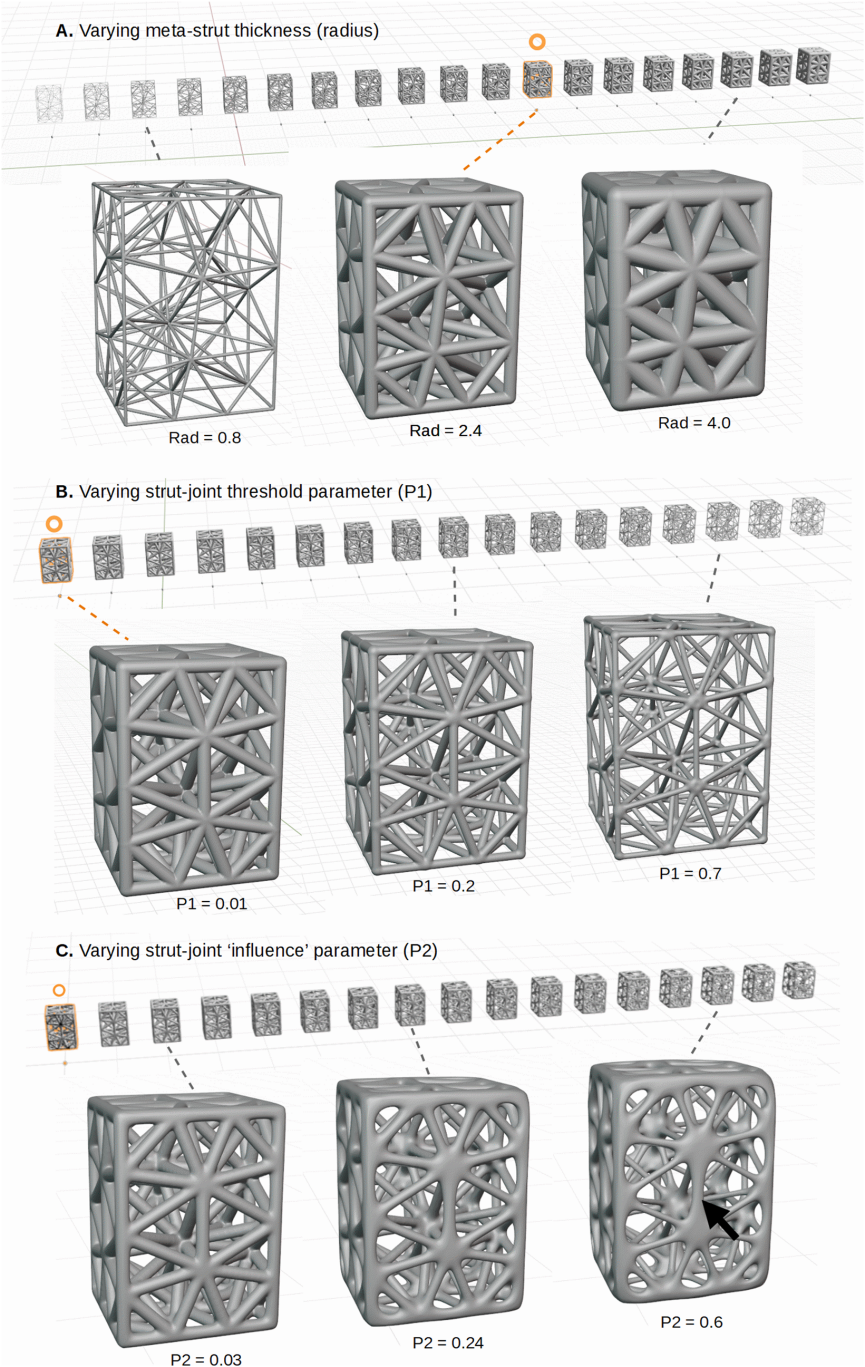
Examples of variations in three main meta-structure parameters: (**A**) strut radius, (**B**) strut-joint threshold, and (**C**) strut-joint tolerance or ‘influence’. Strut radius has the most effect on overall HGon volume change. Threshold (P1) controls the meta-boundary and enables struts to be thinned while retaining joint thickness. Influence/tolerance (P2) controls the blending of struts and joints, enabling enhanced chamfer control. High influence values (> 0.7) can lead to large joints and high tapering of struts (black arrow), which can be interesting for uniquely optimized high strength designs for AM-3D printed components. The circles indicate reference structures for subsequent volume calculations/plots shown in the next figure (Fig. 7).

**Figure 7:**
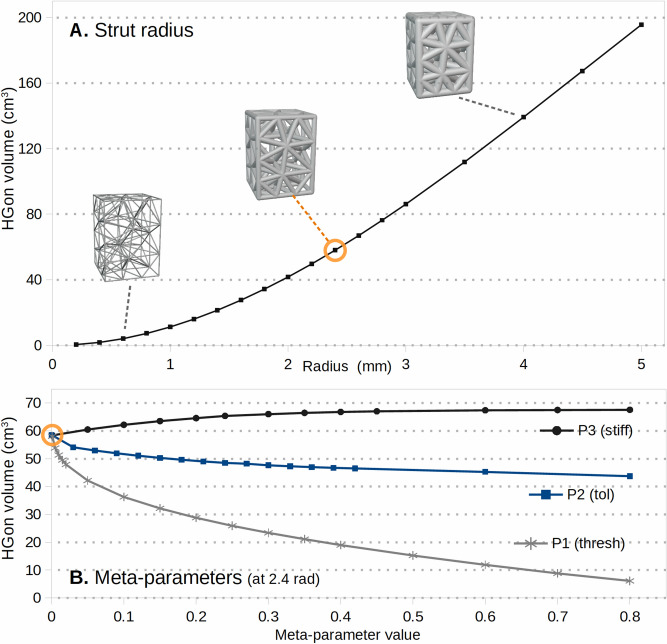
Plots of the variations in total HGon volume versus meta-parameter (**A**) strut radius, which has the most overall effect and (**B**) three other primary meta-parameters: P1-threshold, P2-tolerance/influence, and P3-stiffness. The meta-parameters are computed from a base 2.4 mm strut radius as indicated by the circles (co-indicated in Fig. 6 for reference).

Figure 6 shows examples visually illustrating the effects of varying strut radius and meta-parameters for a simple cuboid (brick). Each meta-parameter varies the structure (radius, chamfer, etc.) in different ways as visible in the figure. Volume changes were also computed varying the strut radius and three of the main meta-parameters; threshold, tolerance, and stiffness (Fig. 7). Further details on the basic process for HGon meta-structure generation are provided in patent (#10585420) and the Appendix A-2. We have tested our algorithm on many complex shapes specifically designed with complex multi-connected topologies. See Appendix A-4 for an example test shape and further description. The test shape geometry and a base (manifold) HGon generated from the shape are provided in the “Supplementary Materials 4” (STL format).

### FEA computational methods

Dynamic FE analyses were used in this 2-part study to compare load-release vibration response of:

- Part 1: A series of simple rectangular lattices and HGon meta-structures.
- Part 2: A multi-connected aerodynamic bracket structure with topology optimization.

The same nonlinear neo-Hookean constitutive parameters and ramp-load-release boundary conditions were used for all structures to ensure consistent comparisons. A neo-Hookean formulation was chosen because of the possibility of large deformations and rotations at lattice and HGon joints. No end ‘sandwich’ masses were used because of concerns that they might artificially ‘flatten’ or otherwise influence the dynamic response. FE calculations were done using standard FE methods for dynamic analyses: FEBio (dynamic, www.Febio.org) and Tochnog (explicit, www.Feat.nl). Details of the FE methods can be found in their respective manuals. All HGon generation, optimization, volumetric calculations, and post-calculations (e.g. stress fields, integrations, 3D interpolations) were done with custom in-house Python (python.org) with CuPy GPU extensions (cupy.dev) and C++ programs.

### Part 1: Analyses of rectangular cuboid shapes (bricks)

**Figure 8:**
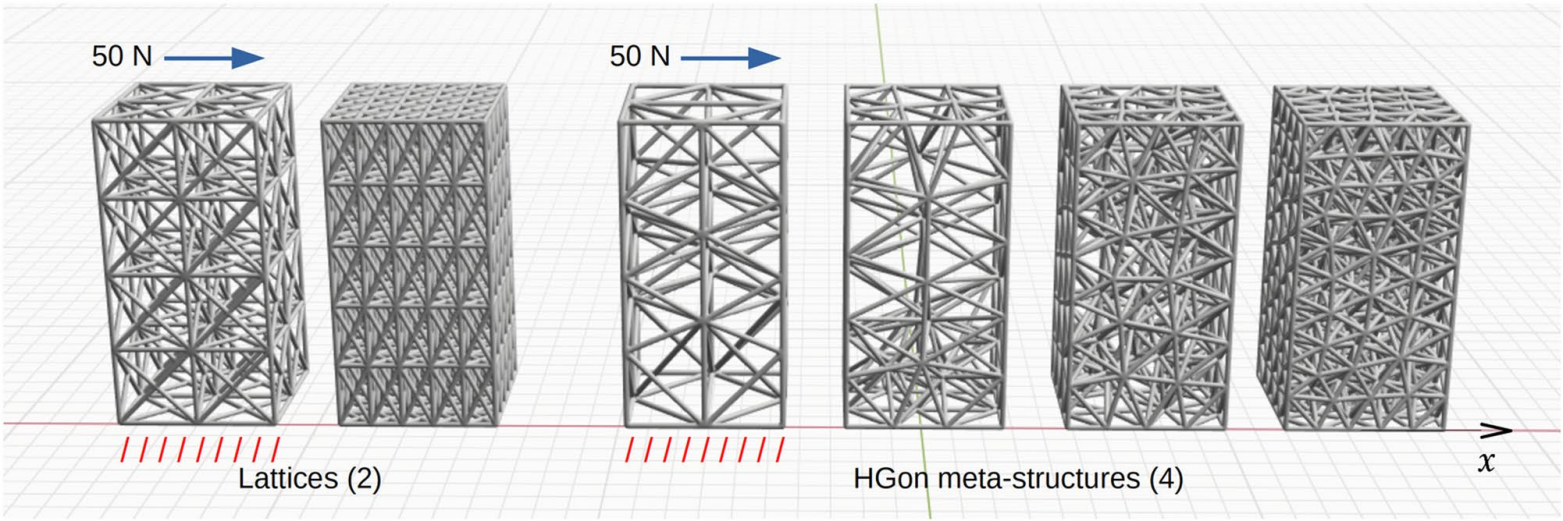
Two rectangular FCC lattices and four HGon meta-structures with increasing density were generated. Each of these structures had exact same applied ramp-and-release loading conditions in the X direction (arrows) and fixed bottom faces as indicated. For the lattices; 4 × 2 and 5 × 5 cells were computed. For the Hgons; 5 × 3, 6 × 4, 8 × 5, and 10 × 6 node densities were computed.

Two rectangular face-centered cubic lattices and four HGon structures (50 × 50 × 100 mm) with incrementally varying densities were generated and analyzed using the exact same ramp load-release boundary conditions (Fig. 8) with nominal strut diameter of 2.0 mm. A 50 N distributed ramp load was applied to the top faces of each structure in the X-direction for 0.1 s, followed by immediate release. Bottom faces were fixed in XYZ for all structures.

The exact same nonlinear neo-Hookean constitutive model and material properties were used for all structures; equivalent to typical AM sintered aluminum material (60 GPa modulus, 0.33 Poisson’s ratio). Preliminary analyses were done to ensure that maximum stresses (< 100 MPa under applied 50 N load) were well below the 170–200 MPa tensile yield stress for this material.

For one lattice and one Hgon, additional 5× time steps were computed to confirm that an initial time step of 0.00002 s was sufficient to capture 10 kHz vibration response. Plots of top total displacement vs. time were computed for all lattices, Hgon structures, and original CAD shell/solids for direct comparison. Due to computing time and memory considerations, FE analyses were done for to two lattice and four HGon structures, each requiring 9–22 h to compute (depending on mesh density) on a dual 4-core CPU (Intel i7, 48 Gb ram) with 100–600 Gb of memory/storage for each structure’s FE dynamic stress and deformation field data. Post-release time step was 0.0001 s for a total time of 0.25 s (or further where possible for the simpler structures). These values were chosen from preliminary studies that achieved convergence for repeated oscillations (target of five full repeats) with sufficient time steps to resolve post-release frequencies up to 5 kHz considering the well known Nyquist sampling limit. An exception was the high density lattice structure that required more time steps (double) to achieve convergence. This was the highest resolution we could achieve in a reasonable time (9+ h/run) with available computational resources.

### Part 2: A multi-connected aerodynamic bracket

**Figure 9:**
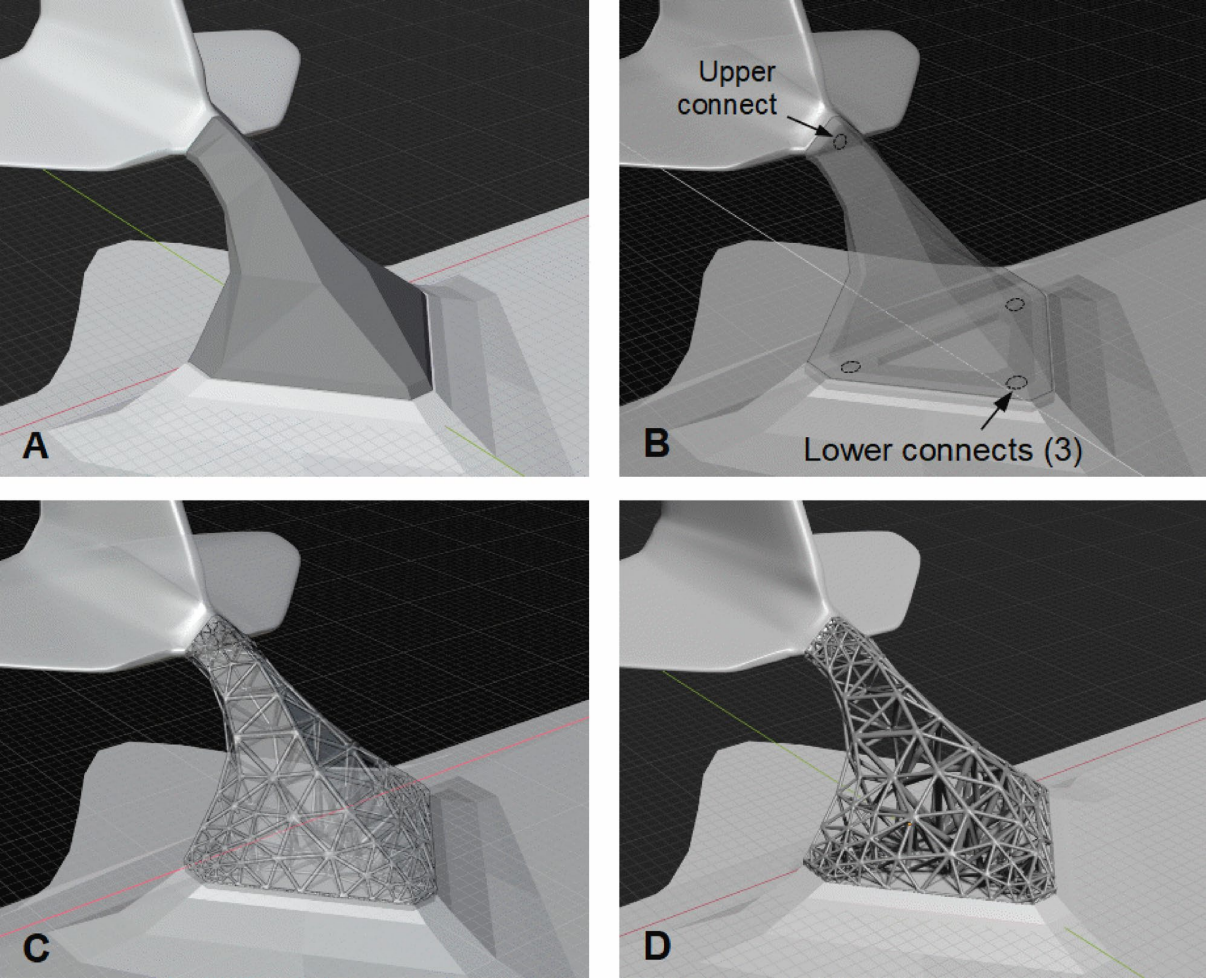
A multi-connected aerodynamic fin-panel support (**A**) original CAD surface/shell, (**B**) connecting holes, (**C**) conforming HGon and CAD superposed, and (**D**) lowest density HGon with minimum 2.4 mm strut diameter. The HGon is fully manifold and AM printed in aluminum or titanium (note the HGon is wrapped in mylar and carbon-fiber membrane for the final component).

A multi-connected CAD model of an aerodynamic bracket-support having both upper and lower connecting holes was first converted to a base meta-structure (Fig. 9). Three conforming HGons with low (28%), medium (52%), and high (70%) volume fraction density were computed having minimum 2.4 mm and nominal 2.6 mm strut diameter. Outer box-dimensions of the bracket were 130 × 230 × 200 mm XYZ, however note that the structure can be scaled to desired size (used with permission).

**Figure 10:**
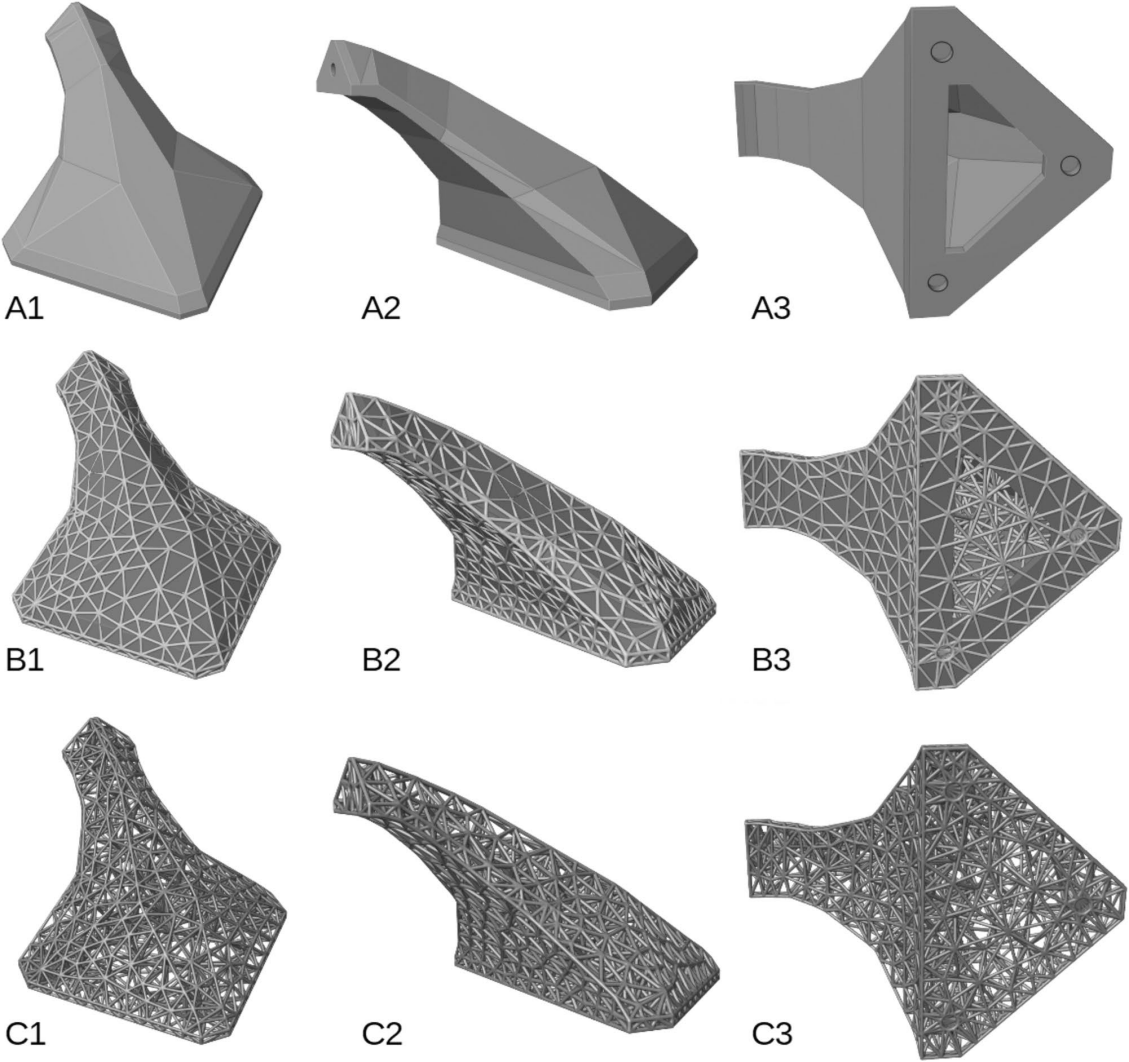
Three views of (**A1–3**) the original CAD bracket structure (**B1–3**) the HGon with CAD structure, and (**C1–3**) the HGon by itself showing the internal meta-structure. Note the tight conformity of the HGon to the original bracket; with edges and mounting holes (**B3**). There are no ‘hanging’ struts or edges as often the case with lattice-type structures. This high-density HGon has nominal 2.4 mm meta-strut thickness and is 42% lighter (volume reduced) compared to the original CAD solid/shell bracket structure.

Conforming HGon meta-structures (Fig. 10) were generated with accurate positioning of connecting surfaces and holes within 0.05 mm required tolerance to the CAD shape boundary. Note that the density around the holes could be more finely graded if needed. Nominal strut diameter was 2.4 mm, thus the overall structure was 1.2 mm larger than the original boundary. To account for this expansion, the CAD file can either be resized (shrunk) prior to HGon generation or in post-generation if desired. Alternatively, since the resulting HGons are fully manifold, *just the connecting surfaces* can be flattened for post CNC machining to meet desired tolerances. Hyper-structure parameters can be assigned individually or globally, such as strut diameter, taper, joint size and radius by either explicit definitions (e.g. sizes at points, edges, or surfaces) or field-based (von Mises stress, strain energy, flow rate, temperature, any other). Joint sizes, chamfers, and tapers can also be optimized to minimize stress concentrations.

**Figure 11:**
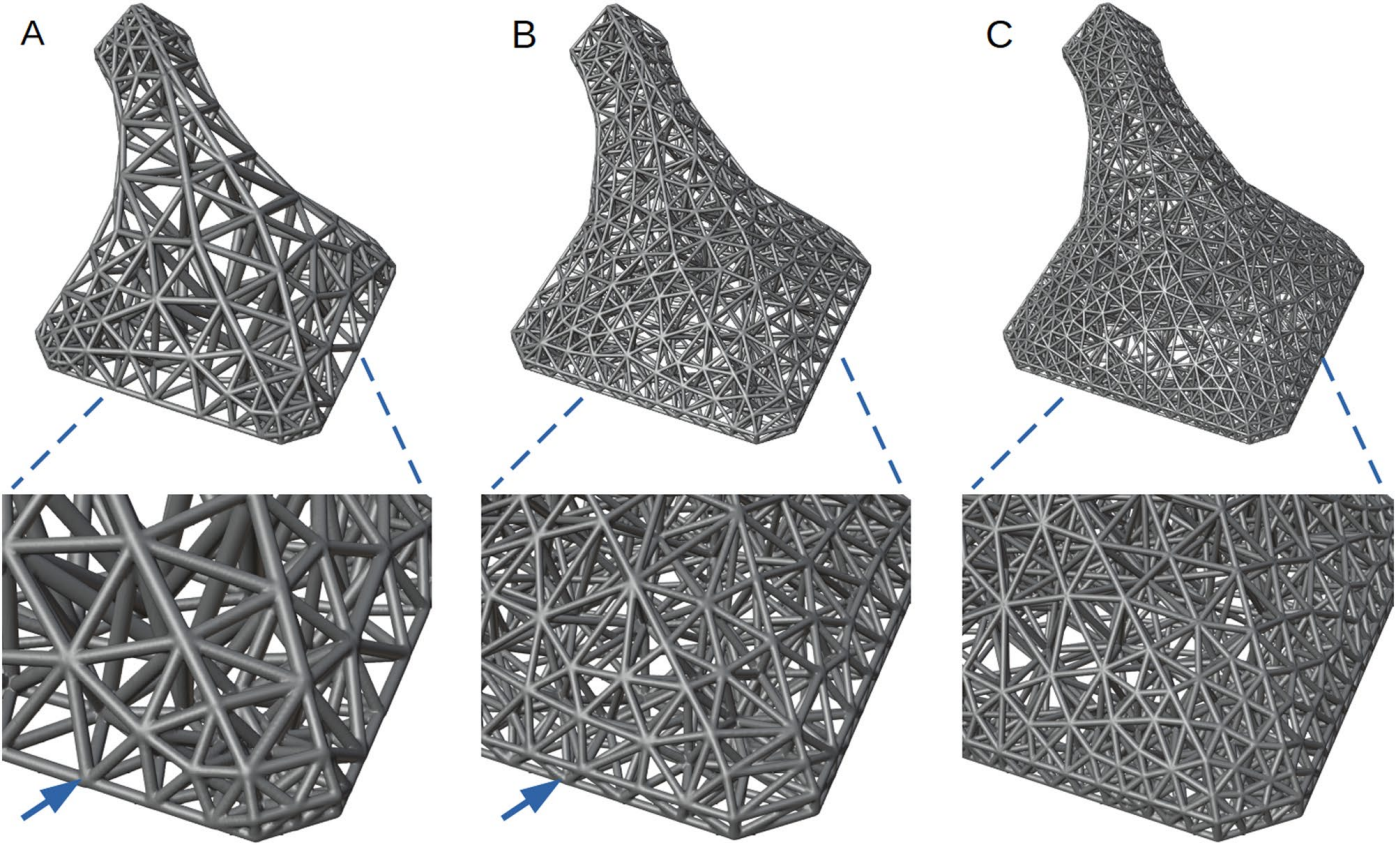
Low, mid, and high density HGon meta-structures. The low-density (**A**) struts are thicker, and the long edge feature consolidated to one lower edge at the bottom boundary (arrows). The mid density (**B**) and high density (**C**) HGons have decreased strut length-diameter and hence capture finer detail.

All HGon meta-strut lengths, thicknesses, tapers, and joint radii are parametrically controlled int the optimization to improve strength or reduce stress concentrations while maintaining printability and essential CAD surface details. Note that there are always trade-offs, as visible in the lower boundary (Fig. 11, arrows) where a lower edge is consolidated or ‘lost’ for the low density HGon, but retained in the higher density HGons (Fig. 11B–C). How much detail is retained in surface definition is often a matter of external factors and user design criteria. For instance, the ‘lost’ edge shown in Fig. 11A is not an essential feature for this aerodynamic structure, thus the thicker/stronger HGon is preferred in this particular design case.

#### Pre-FEA and meta-meshing

Preliminary FE analyses were performed to compute a ‘base’ HGon meta-structure having nominal density and strut diameter (2.4 mm) to achieve required 2.0 factor of safety. The primary structural criteria was that deflection of the narrow end should be less than 1.5 mm under 100 N applied Z-load. Secondary criteria were also included such as AM processes and material(s) to be used.

**Figure 12:**
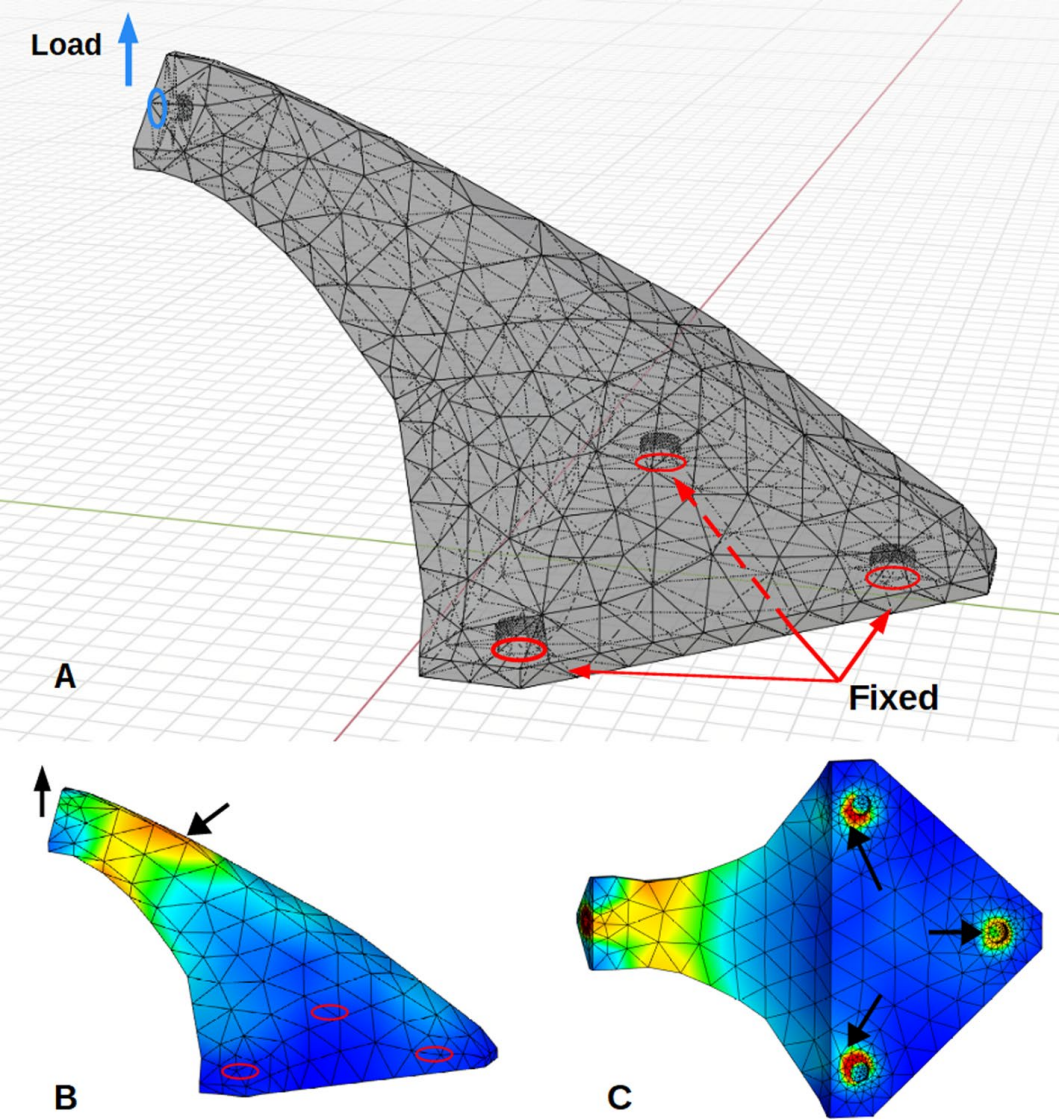
(**A**) boundary conditions for all analyses showing the upper connector applied load (+ 100 N) and three fixed connection holes. Views (**B**, **C**) show preliminary-computed stress fields (von Mises) with arrows indicating locations where highest total displacement and stress vs. time were plotted in the results.

Boundary conditions (Fig. 12) were the same for all preliminary and subsequent analyses of shells and HGons with three fixed base connections and Z-axis loaded upper connection.

The initial ‘base’ HGon meta-structure is generated with baseline mass/density to support sufficient upper-bound peak stresses (component strength) with the desired AM material (in this case sintered aluminum) and conformation to boundary/connecting surfaces. Due to NDA considerations we cannot describe all details here, but additional conditions and criteria include the desired AM process (e.g. powder-bed SLS, resin SLA, electron beam, FDM-type, etc.) and post machining of the AM-3D printed meta-structure connecting surfaces. Note that all parameters can be varied to accommodate a wide range of AM 3D printing systems, with primary constraints typically being the minimum strut thickness, overhang angles, and joint thicknesses. Also, HGon structures can be *superposed* or otherwise combined to achieve multi-material and multi-density structures enabled by new AM jet and multi-powder printing systems.

**Figure 13:**
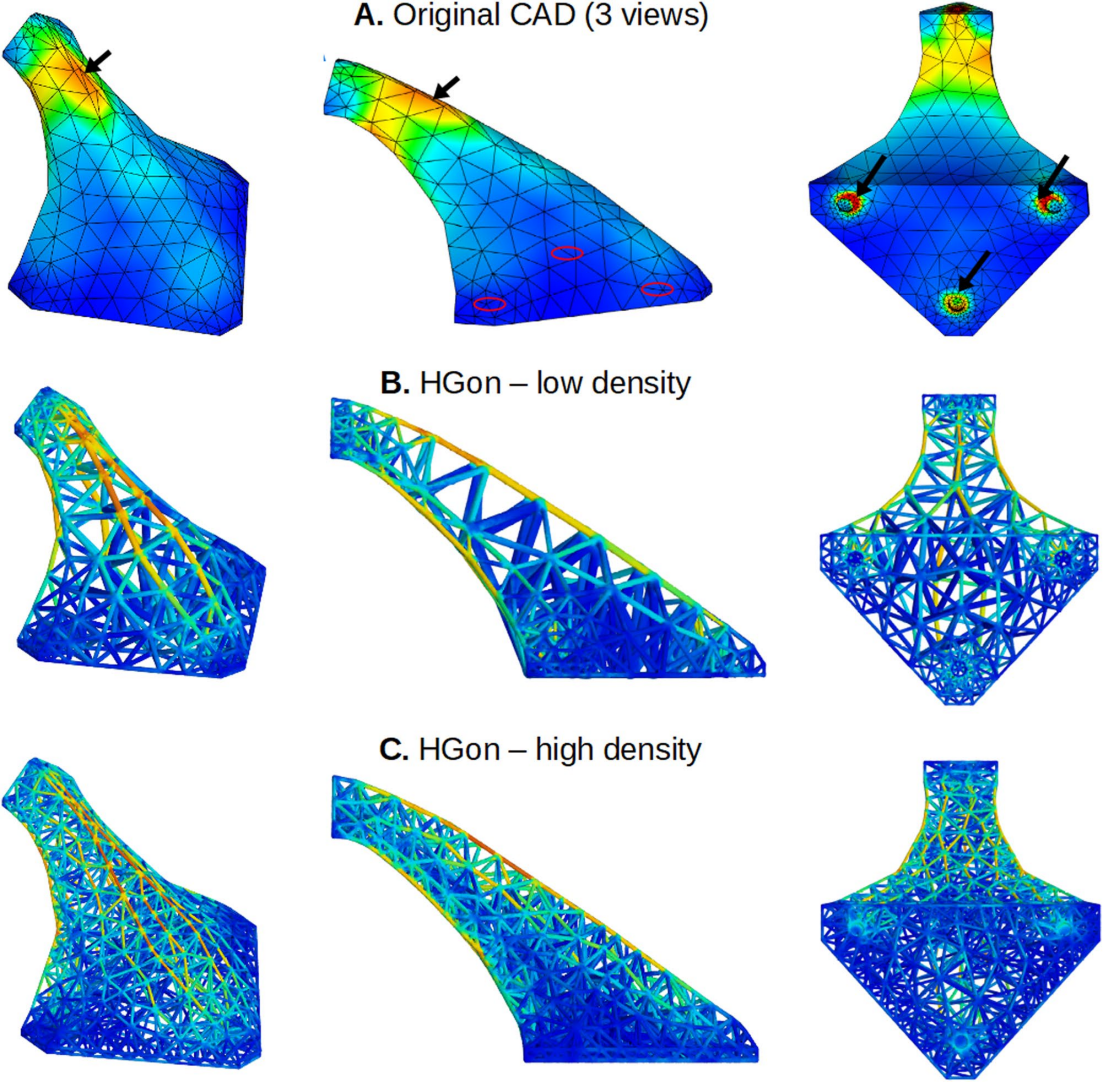
Three orthotropic views (oblique, side, bottom) of the original CAD (**A**) and the three HGon meta-structure FE meshes (**B**–**D**) showing maps of von Mises stresses (0–70 MPa range) at peak (pre-release) load. The black arrows in (**A**) indicate locations of high stress used for the stress-time plots in subsequent figures.

Tetrahedral meshing of the *original* CAD solid/shell is quite easily done using a number of available codes (e.g. Netgen: ngsolve.org, Gmsh: gmsh.info). However, these meshing codes do not apply well to HGon meta-structures, largely because of joint topology complexities. To address this and related issues, we developed a custom HGon meta-mesh generator that produces well-graded and high angle tetrahedra and hybrid tetrahedra-hexahedra for HGons and similar lattice meta-structures. The custom mesh generator enforces smooth joint-strut transitions and fully automated removal of ‘slivers’. Slivers^24^ are an important issue when generating these HGon-type structures as they can result in undesirable joint morphologies. Slivers should be minimized or at best completely removed. In previous work we have detailed our novel, heuristic, multi-manifold sliver-removal process^25^. The new HGon meta-mesh generator successfully achieved 100% sliver removal for all three HGon meta-structures presented here. Figure 13 shows three views of the original CAD and three generated HGons. FE meshes show full conformity to the complex surface and smooth sliver-free FE stress field calculations.

This pre-FE analysis was also used to confirm that there were no undesirable discontinuous or non-manifold features in the base or optimized meshes. Stress distributions were examined to ensure the analysis produced results that were consistent with cantilever-type boundary conditions of 100 N + Z axis load. Computed stresses were all smoothly graded (no apparent singularities) with highest stresses at the upper and lower surfaces of the structure. Stresses were also elevated at the front lower connectors compared to the rear connector, as would be expected from this cantilever-type configuration.

**Figure 14:**
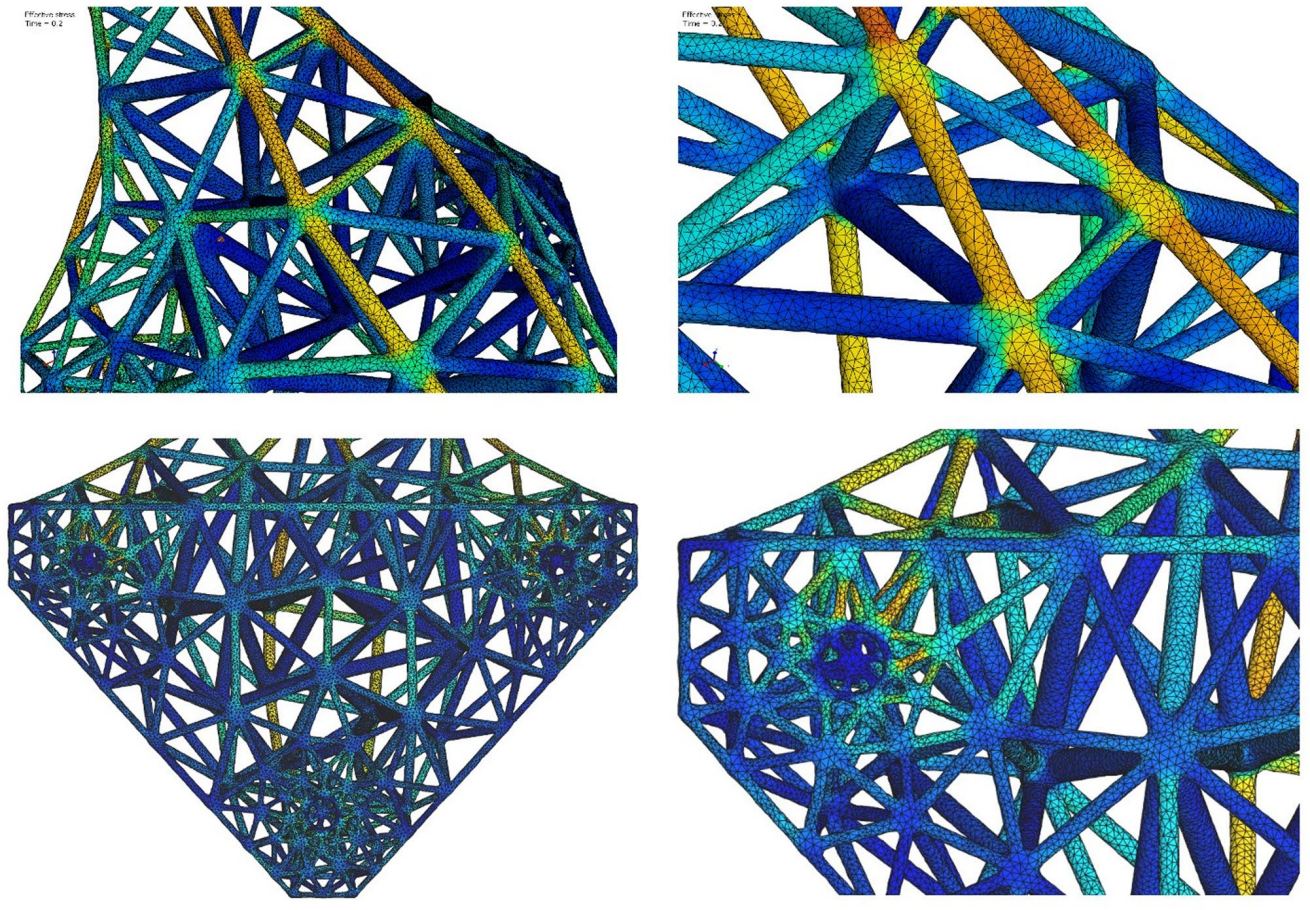
Four closer views showing detail of the lower-density HGon from Fig. 13B. Note the well-shaped tetrahedral elements with smooth topological transitions between the joint and strut structures. Initial computed von Mises stresses are also smooth, with no ‘jumps’ or concentrations that would indicate slivers or other undesirable FE mesh characteristics. The HGon accurately captures the multiple hole connectors, curved and straight edges, and other features of the original CAD solid.

Close-up views (Fig. 14) of the HGon meta-mesh structures show well-shaped tetrahedral elements conforming tightly to the CAD and HGon structure. The HGon FE meta-mesh generator accurately captured both the straight and curved CAD edge boundaries and connecting hole features, with smooth transitions between sharp-to-round boundary structures. Transitions are also well-graded from the boundary to interior of the structure. Slivers were 100% removed. Because meta-meshes are fully automatically computed, all parameters can be varied and controlled programmatically to enable a wide range of optimization strategies.

#### Field-adaptive stress optimization

HGon meta-structures can be stress-optimized, with global/local optimization/adaptation of meta-density and strut/joint thickness among other topological features. For this study only the first loading ramp (peak) of the simulation was used for stress optimization (von Mises stress). Further vibration cycles, modal vibration calculations, or any other computed fields and also be used to drive the HGon meta-structure adaptation process.

The meta-topology optimization procedure is summarized in the following four steps.

1. *Initial FE analysis is performed with proscribed load and fixed boundary conditions.*
  For this bracket, three fixed connectors are located on the lower wider face (see Fig. 10) and the single connector on the upper face has applied Z-axis load of + 100 N.
2. *Stresses (and/or other fields) in the base meta-structure are computed (von Mises).*
  Computed fields are discretized and exported as high-density (4× over-sampled) point-wise volumetric data. Note that *any* field, or combinations of fields can be computed and used for this step via this approach, which is newly possible with latest GPU/RTX ray-tracing volumetrics and vector engine technologies.
3. *HGon meta-structures (e.g. strut and joint thickness) are then topology modified.*
  Meta-structure radii and joint size are ‘thickened’ or ‘thinned’ by (currently) a linear ratio of the computed local stress from step 2. Similar can be done for other topological features (e.g. joint taper or chamfer).
4. **Repeat** steps 2 and 3 until overall and local stress reduction criteria are reached and/or the resulting iterated topology changes are minimal (in this case < 5% deviation).

**Figure 15:**
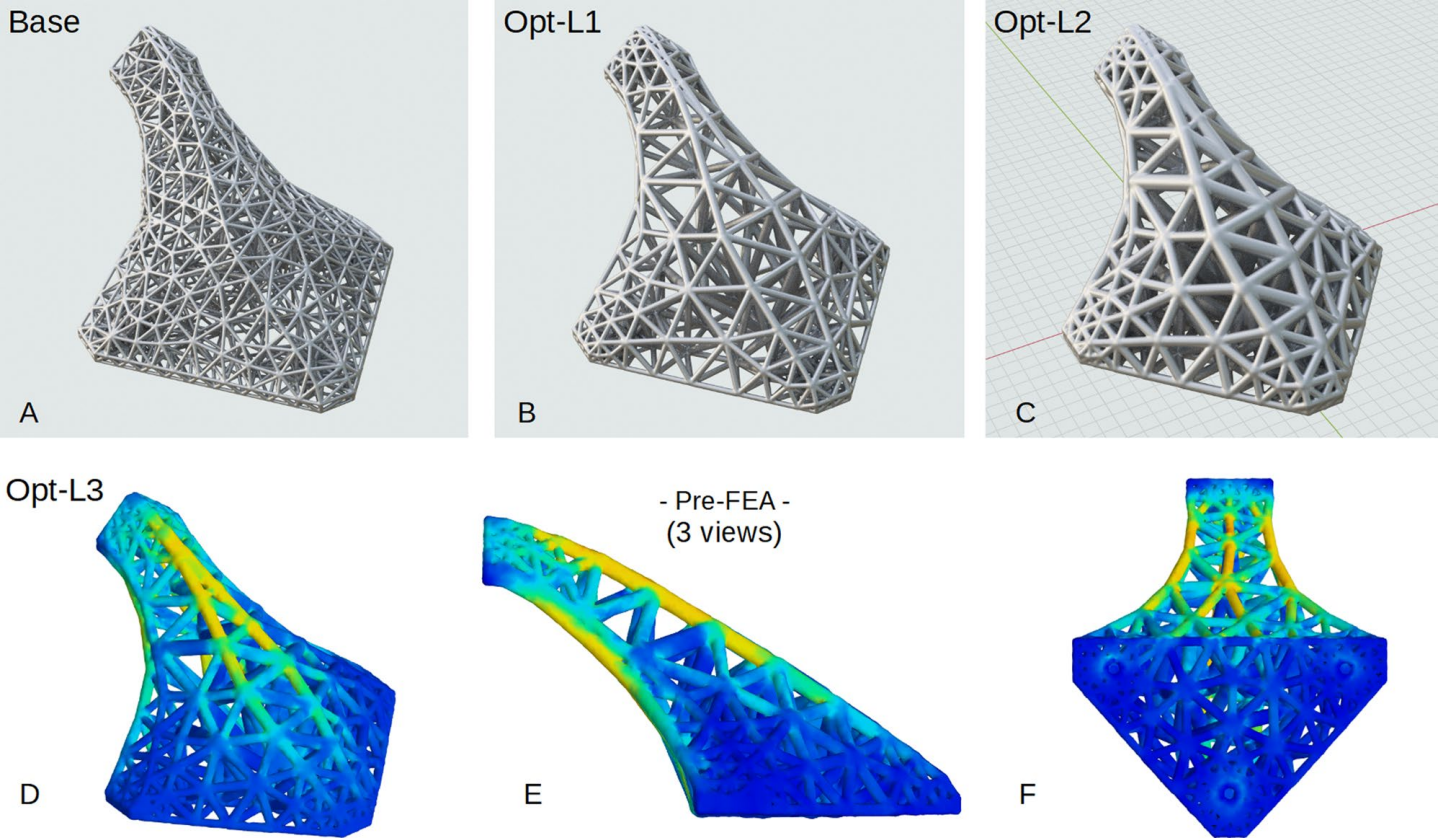
Stress optimized meta-structure starting with (**A**) initial ‘base’ HGon, (**B**) first density optimization, (**C**) second iteration, and (**D**–**F**) three views of the L3 final iteration. Struts and joints are thickened, thinned, or removed in accordance with stresses computed from the pre-FEA with for this study a simple + Z axis 100 N applied load. Other loading combinations can be applied which would result in a different final HGon adapted/optimized structure.

The ‘base’ HGon and stress-optimized structures computed using this 4-step procedure show stress-adapted meta-struts thickened in the upper and lower high stress regions (Fig. 15), achieving the optimization goal of increased support for the applied + Z axis load. This effectively stiffened the HGon in the Z-direction. When strut stress was below a threshold (in this case < 2 MPa), the entire strut was procedurally removed. FEA results show reduced stresses in all struts and connectors (Fig. 15D–F).

This 4-step process can be extended to multi-axis loading conditions, which would of course result in a different optimized structure. We note that this is a different approach than is normally used for surface-based topology optimization. For this application it was required that that surfaces be constrained for aerodynamic and post-printing considerations. Also, for this study only von Mises stress to drive the meta-structure optimization. Additional computable fields such as strain energy, shear stress, cumulative stress (fatigue), heat transfer, and electromagnetic effects can also be computed and used individually or in combination.

#### FE vibration analyses: complex bracket

Quasi-static and dynamic FE analyses were performed for the original CAD-derived solid/shell structure and the three HGon meta structures. The same boundary conditions (see Fig. 12) were applied for each structure; a 100 N ramp load (Z-dir) followed by immediate release. Thus the Hgon bracket was allowed to vibrate freely; no extra dampening masses were added. Nonlinear elastic neo-Hookean material properties were used for all analyses representing sintered aluminum: modulus = 60 GPa, Poisson’s ratio = 0.33, and density = 0.0027 g/mm^3^. To simulate damping effects, a viscoelastic term (generalized Maxwell type) was added to the constitutive model, identically for all analyses.

Ramp load was applied in 0.2 s with dynamic calculations proceeding using a time interval of 0.00002 s up to a minimum of 0.25 s (2500 steps). Because of computational and large data storage requirements, not every time step was stored for the HGon meta-structures, which often had over 2 M elements and required large 500–2000 GB storage per calculation. However enough time steps were stored (at typically every 2nd–4th step) to accurately represent and plot/compare the vibration waveforms.

Typical time for one run at high HGon FE meta-mesh size (2.3 M elements, 2 K + steps) was 22 h (2 × 8 core Intel i7, 48 Gb ram). Matrix inversion and LU decomposition was done using a customized version of the Pardiso solver (www.pardiso-project.org) that enabled CPU specific optimizations. FEA run time for the stress-optimized HGon (reduced to ~ 700 k elements, 2 K steps) typically was 2–3 h. Note that several runs (6–12) were performed on both lattices and HGons with increasing element densities to confirm convergence.

Total displacement vs. time was computed and plotted for the original CAD and HGon structures for an element located directly above the upper connector where load was applied. This was the location having highest total displacement. Plots of von Mises stress vs. time were also computed for four selected elements, one at the top mid-strut location that experienced maximal stress variation, and three elements at the bottom face connections (see Fig. 12C). Videos of overall dynamic stress and deformation responses were generated for all shell/solid and HGon structures (please see the “Appendix”).

## Results

### Part 1: Rectangular structures

**Figure 16:**
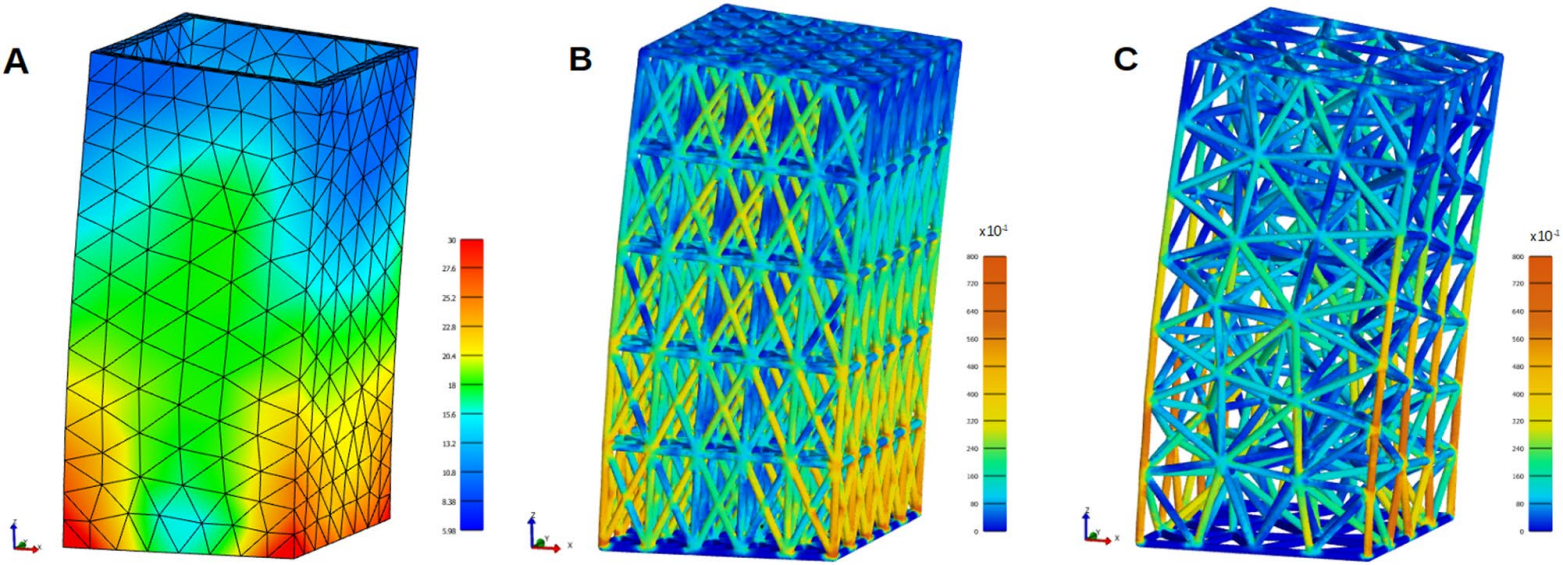
Bending stress (von Mises) and deformation of (**A**) the original solid/shell, (**B**) a high density lattice, and (**C**) a mid-density HGon (8 × 5). Note the high stress concentrations at the lower lattice (**B**) base joints which do not appear in the HGon meta-structure. Deformations are magnified 5× for visualization.

Computed deformations and FE stress distributions at peak load showed that the structures all behaved in standard cantilever fashion (Fig. 16). Stresses were lowest in the solid/shell, which was expected. Highest stress concentrations were found at the base of the lattice structures, but were reduced for the HGon structures. Strut stresses were also lower for the HGons compared to lattices. With the exception of the lattice base-corner joints, all stresses were well within the yield criteria for sintered aluminum.

**Figure 17:**
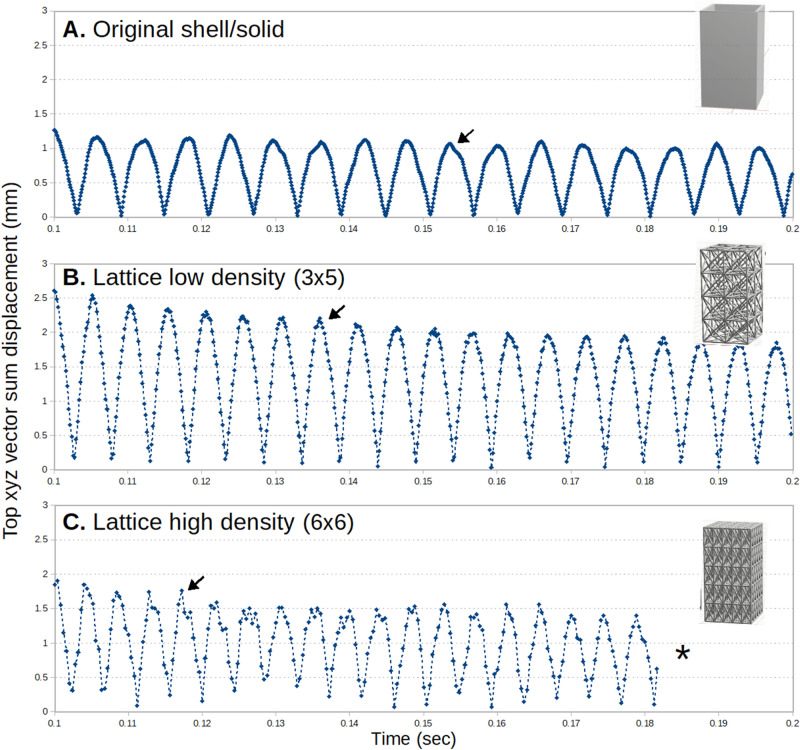
Maximum *top* (xyz vector sum) displacement vs. time for (**A**) the original shell structure with 2.5 wall thickness and (**B**–**C**) standard lattice structures. Arrows indicate high frequency responses at peaks which are more evident for the lattices. *Note, in (**C**) the data is truncated at 0.182 s due to available system memory limitations.

Plots of xyz *vector-sum* (total) top displacement vs. time (Fig. 17) show the differences in response for the shell and lattice structures. Note that vector-sum combines displacements in all three directions and is a magnitude (R > 0). For further description and an example plot of X-direction displacement (oscillation) please see the Appendix A-5. Vector-sum was chosen to best and most succinctly represent the full displacement. Since these are 3D structures, small oscillations in the Y and Z (mostly Z) axes also contribute to the overall displacement. Thus the vector-sum top displacement is a more representative measure of overall structural response.

The shell structure was approximately 2× stiffer than the computed FCC lattices and showed minimal damping for the duration of the calculation. The low density lattice vibration response showed a small damping effect. The high density lattice was the stiffest with highest frequency response and had more erratic vibration patterns.

**Figure 18:**
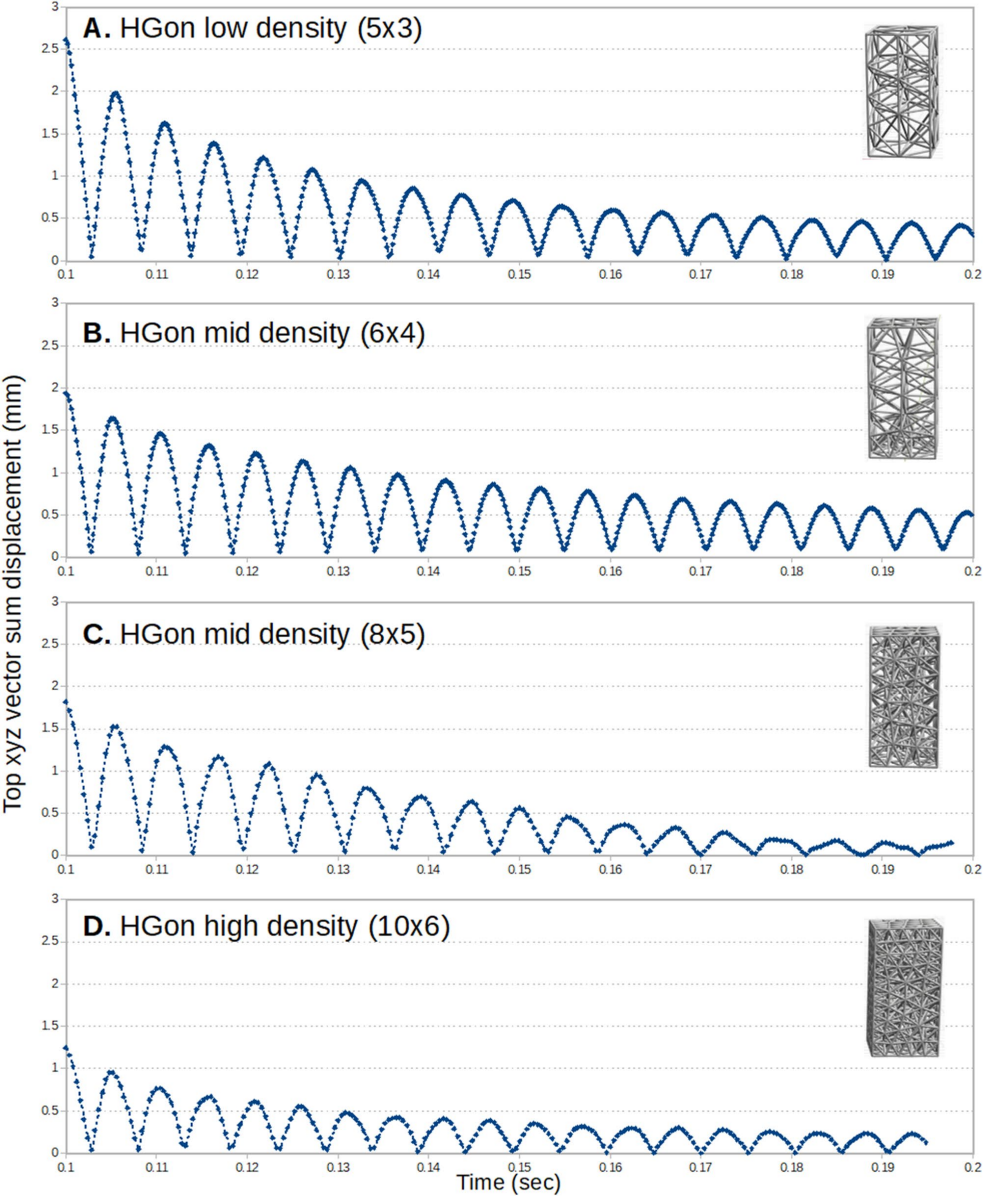
Maximum displacement versus time for the rectangular HGon structures with four incrementally increasing densities. The highest density HGon (**D**) was the stiffest and had shortest period of vibration, while the 8 × 5 mid-density HGon (**C**) appeared to produce the strongest dampening effect overall. Vibration response overall was much smoother for HGons than for the lattice and shell structures.

Plots of vector-sum top displacement vs. time for HGon structures (Fig. 18) showed near total elimination of the high frequency components and higher damping effect (note that all plots are raw, unfiltered). The mid-density 8 × 5 HGon (Fig. 18C) produced the highest damping effect, with nearly all of the vibration response dampened by the end of the calculation.

The highest density HGon (Fig. 18D) was the stiffest of all structures, approximately 2.2× stiffer than the low density HGon, also having the shortest period of vibration. HGon masses from lowest to highest were 14.2, 22.1, 30.5, and 52.1 g, respectively. Note that the original rectangular shell mass was 74.4 g (aluminum-type material).

### Part 2: Aerodynamic bracket results

**Figure 19:**
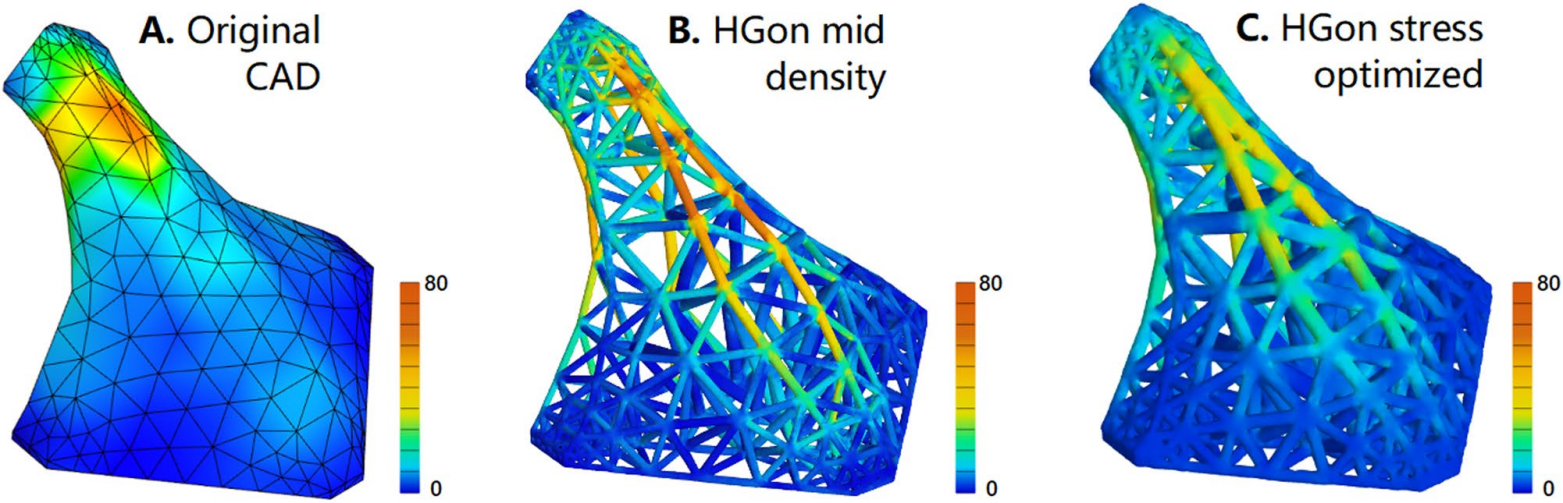
von Mises stresses computed for (**A**) the original CAD shell/solid, (**B**) HGon meta-structure mid-density, and (**C**) the stress-optimized HGon. Stresses in the optimized HGon were reduced up to 60%.

FE computed stress results were realistic and consistent with bending for both the shell and HGon meta-structures with highest stresses appearing at the upper and lower regions of the solid/shell CAD surface and in similar regions for all HGon meta-structures (Fig. 19). Maximum computed stresses were 72 MPa for the CAD solid/shell, 85 MPa for the mid density HGon, and 46 MPa for the stress-optimized HGon. All stresses were well below published yield stress of 153 MPa for aluminum-type materials in AM sintered processes^26^.

Volume of the original complex CAD shell/solid structure was 71.4 cm^3^. HGon meta-structures had reduced volumes of 12.8, 18.6, and 44.8 cm^3^ for the low density, mid density, and stress-optimized structures, respectively. HGon meta-structures thus had volume reductions of 72%, 54%, and 37%. Masses of generated HGon structures (aluminum) were 35.1, 45.2, and 121.0 g compared to 192.8 for the CAD solid/shell.

**Figure 20:**
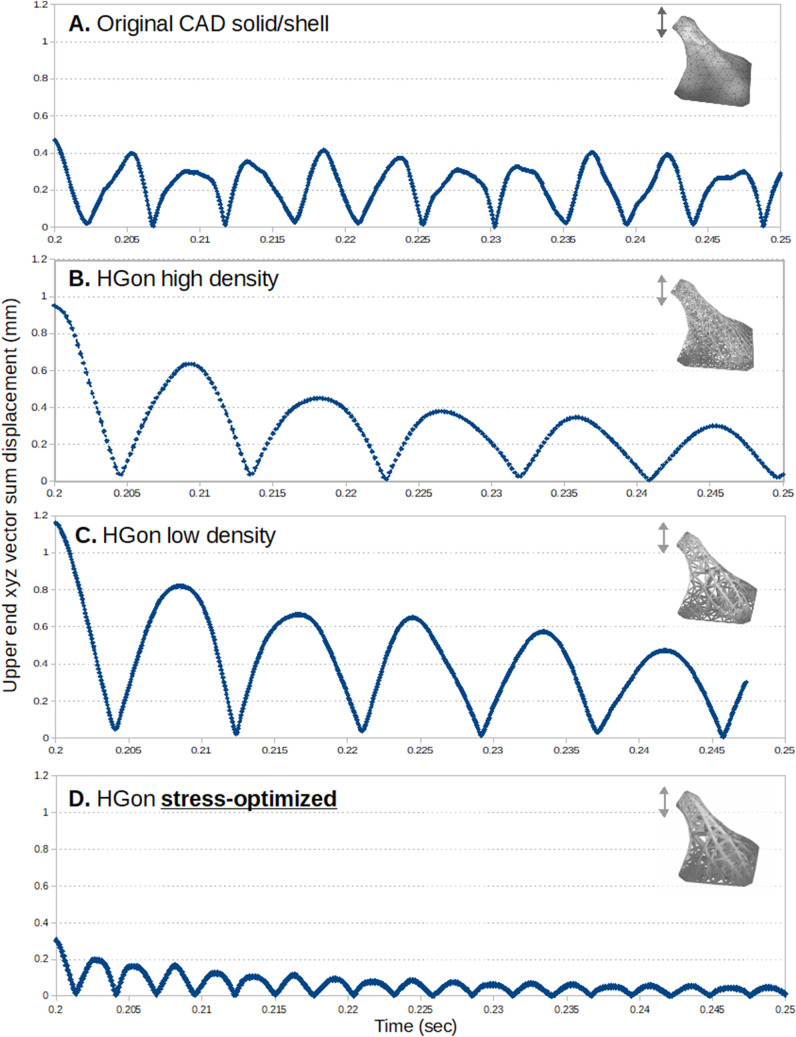
Upper end displacement–time plots for (**A**) the original CAD solid, and (**B**–**D**) the generated HGon meta-structures. The CAD shell showed little or no damping within the time period of analysis, whereas the HGons displayed damping behavior. The stress-optimized HGon (**D**) was the strongest (stiffest) of all structures with correspondingly higher vibration frequency.

Upper-end displacement vs. time response (post-release) for the original CAD solid/shell showed complex repeating patterns, irregular waveforms, and visible high frequency components (Fig. 20A). Little or no damping was visible for the shell/solid.

In contrast, HGon meta-structures showed reduced high frequency vibrations (Fig. 20B–D), longer periods of vibration and much higher dampening characteristics. This reduction of high frequencies was surprisingly large and similar to the results from the simple rectangular structures in Part-1. The stress-optimized HGon had highest stiffness; 0.35 mm max displacement compared to 0.44 mm for the CAD shell/solid.

**Figure 21:**
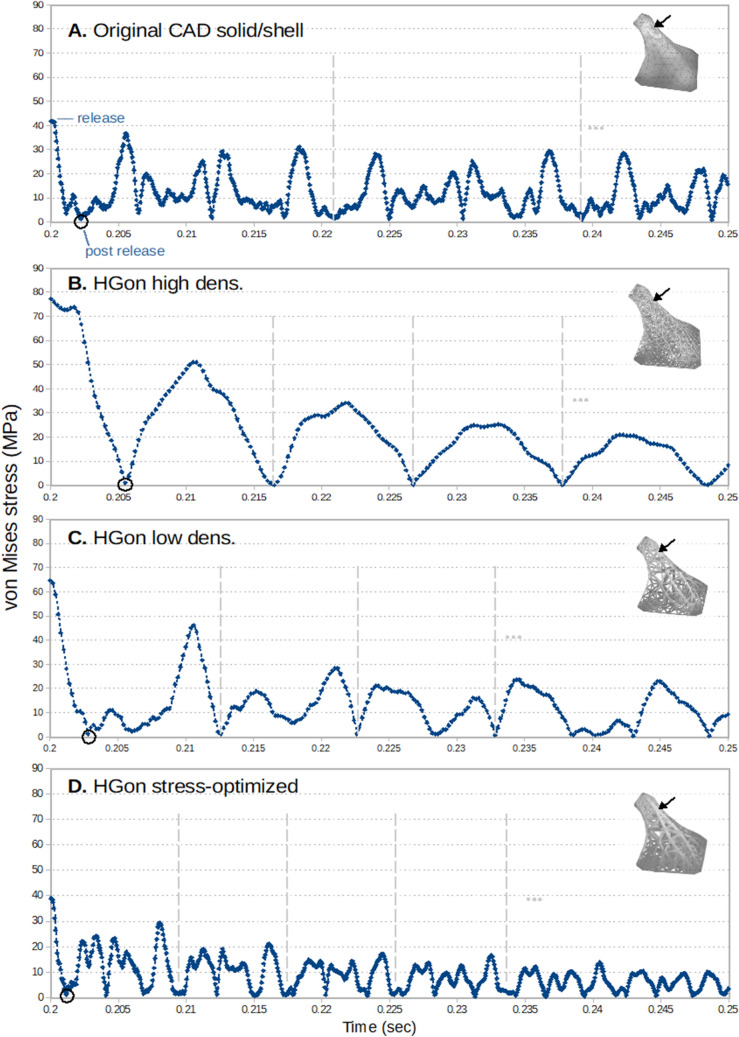
Plots of von Mises stress (MPa) vs. time (s) post-release for (**A**) the original CAD and (**B**–**D**) the computed HGons. Black arrows indicate the locations of plotted (highest stressed) elements. Black circles indicate end of post-release. Vertical dashed lines indicate repeating patterns. The stress-optimized HGon (**D**) was the strongest overall both for peak stresses and showed strongest dampening behavior.

Von Mises stress vs. time response at the *top* location of the CAD solid/shell (Fig. 21A) showed little damping and again high-frequency response. HGon meta-structures showed much higher damping and smoother response for both high and low density meta-structures (Fig. 21B–C), The stress-optimized HGon (Fig. 21D) was clearly the strongest overall with lowest peak and average stresses. Repeating vibration patterns are indicated in the figure by the vertical dashed lines.

**Figure 22:**
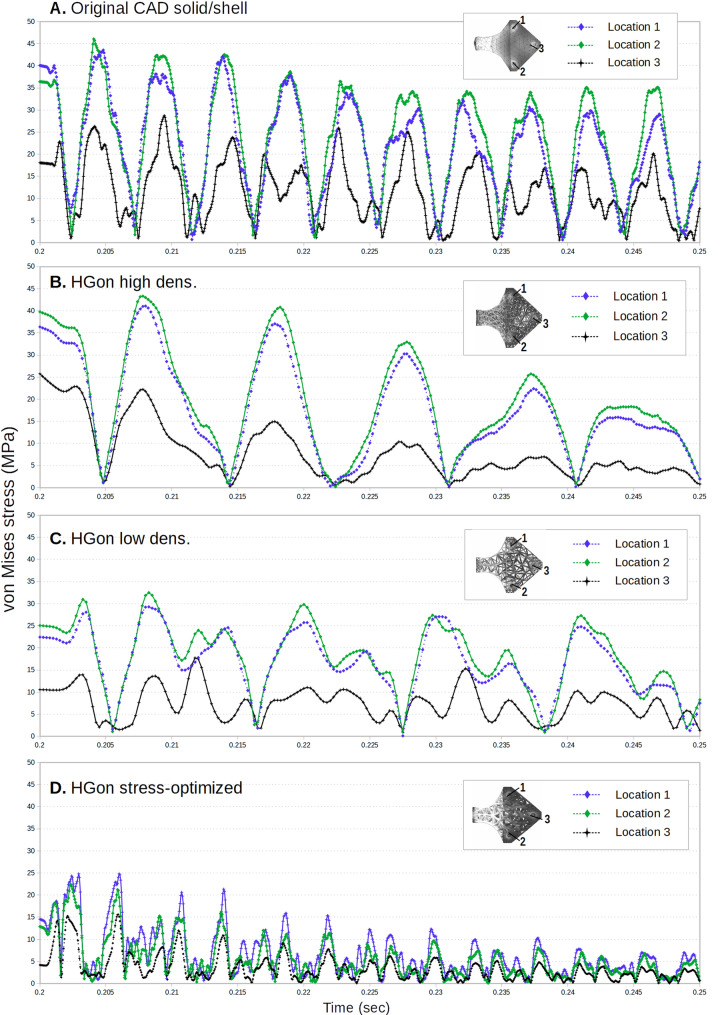
Plots of von Mises stress (MPa) vs. time (s) at the three connections as indicated. Stress was highest at the two front locations (1 and 2) for all structures. HGon results show reduction of peak stresses and dampening of higher frequency vibrations compared to the CAD shell/solid; in particular for the stress-optimized HGon meta-structure (**D**) which was the strongest overall, even stronger than the original shell/solid.

Plots of von Mises stress vs. time for the three bottom connection locations (Fig. 22) showed high frequency components which were strongly damped in the HGon meta-structures. Stresses at the two rear connectors were much lower (typically < 50%) than for the two forward connectors. The stress-optimized HGon (Fig. 22D) is stiffer and thus had higher frequency response compared to the lower density HGons. Optimized HGon stress was reduced by ~ 50% up to 70%.

## Discussion

New AM (3D printing) capabilities enable production of meta-components with topologies that were not previously possible using standard manufacturing methods. HGon meta-structures can provide unprecedented strength-to-weight ratios and vibration dampening capabilities, improving on standard periodic lattice structures. However, there are no studies (to our knowledge) that directly analyze and compare these types of complex structures using the same applied dynamic FEA ramp-release loading conditions, boundary conditions, and material properties. FE analyses can provide valuable insights into the dynamic behavior of these and related meta-structures.

This study presents a series of dynamic FE analyses comparing original CAD solid/shell(s), standard lattices, and new *conforming* HGon structures with varying densities and stress-optimization. To achieve robust meta-mesh generation for these complex topologies, a custom hybrid tetrahedral-hexahedral mesh generator was developed. Novel data management strategies were designed to manage large element and property variables, often having up to 3 million elements and > 12 properties per node/element. File sizes often exceeded 2T-Bytes for just one component stress-time dynamic analysis. These are the largest we can computationally manage at this time, but it is certain that even these file sizes will be considered small for near-future computational resources.

Results of end-deflection and peak von Mises stresses supported both the hypotheses: (H1) HGon meta-structures reduced high-frequency response compared with lattices, and (H2) increased dampening was found for HGon meta-structures under ramp-and-release loading conditions. Notable is that high frequencies clearly visible in the solid/shell and lattice responses *were essentially eliminated in all HGon structures.* This result was surprising, as we did not expect the HGon vibration suppression effect to be so large. Animations of the response of the structures (see “Appendix”) provide additional insights into possible mechanisms for this enhanced damping; namely that the non-repeating meta-topology of the HGon struts and joints could be providing a “canceling-out” effect as compared to lattices that have periodic structures with mostly uniform strut orientations and joint structures.

A major benefit of HGon meta-structures is that they are locally (and automatically) conforming to most any CAD surface topologies, even those with holes and/or multiply-connected features. There are no ‘hanging’ or loose structures as often the case with lattice-type periodic structures. HGon meta-structures can simultaneously accommodate strongly curved and straight edges, holes, multi-connectivity, and other topological complexities that standard lattices cannot. HGons are also not limited to tetrahedra—multiple hybrid meta-structures with hexahedral, octahedral, and other polygon ‘primitives’ can be used and combined depending on the application and component requirements. For example, hybrid meta-structures can be parametrically generated to enable guided porosity and strength-density such as for bioengineering or fluid directed structures. Generated HGon meta-structures are fully manifold and printable using standard AM methods. 3D model data for selected HGon structures generated for this study are given in the “Appendix”.

HGon meta-structures achieved over 2.2× of the required strength criteria, even with weight/mass reductions of *up to 72%* compared to the original CAD solid/shell. The stress-optimized HGon was (again surprisingly) the strongest structure overall, even compared with the original CAD solid/shell. However, the stress-optimized HGon also vibrated faster with a shorter wave period. This demonstrates that there are trade-offs between overall structural stiffness and vibration response. Work is ongoing to investigate new multi-material, multi-domain and gradient-material HGons that are even now 3-D ‘printable’ using newly available multi-material AM systems such as the latest HP Jet Fusion, Stratasys, and EOS systems. Because HGons are open framework meta-structures, they can also be infiltrated with soft materials such as silicons, ballistic gels, or foams. This can even further enhance HGon strength and vibration dampening capabilities as well as provide improved fatigue resistance.

Time-series videos of the rectangular and complex HGon vibrations (see “Appendix”) reveal further insights into the meta-structure behavior. Individual struts can be seen to vibrate back-and-forth essentially perpendicular to the strut’s orientation. Thus, the individual internal meta-struts are dissipating energy. Since HGons are overall isotropic by design, this dissipation for HGons is distributed semi-randomly throughout the structure, rather than being directed through as for the periodic lattice structures. This could explain in part why HGon meta-structures show enhanced vibration dampening compared to standard lattices. However this is only one observed factor in this initial study; further study is needed to more comprehensively quantify potential effects of other possible dampening effects of meta-structure parametric topologies such as optimizations of joint masses and strut chamfers/tapers.

Results show that HGons can provide ‘tune-able’ vibration control. By varying HGon densities and thicknesses (parametrically controlled), vibration frequency responses and damping can be both locally and globally controlled and optimized to achieve desired component performance criteria, such as damping certain frequencies or frequency ranges. The response plots (Figs. 15, 16) clearly show the HGon vibration period shortening with increasing strut thickness and Hgon density. HGon parameters can thus be ‘intelligently’ adjusted to achieve multiple goals; e.g. vibration response plus strength-to-weight criteria. Another surprising result was that high stress concentrations visible in the base of the lattice structure and joints were strongly reduced for equivalent density/strength HGon meta-structures. This implies that HGons can enable significant improvements in failure and fatigue properties compared to standard lattices. This is a focus of future work with new experimental testing system in development.

## Limitations and future work

This study only begins to investigate the potential of HGons for vibration response. The goal here was to start with simple cuboids and introduce a new meta-structure and dynamic FEA computational procedures, with first comparisons to lattices and example topology optimizations. For this study we used only one loading condition, applied for each structure (X or Z-axis ramp) and a relatively small (< 0.3 s) time range due to computational limitations. Future extended analyses are planned with multiple loading conditions, longer analyses times/resolutions, and wider parametric ranges as computational resources become available.

In Part 1 the analyses were limited to six structures (2 lattice, 4 HGon) which in total required ~ 1800 h to compute. However, large speed improvements can be obtained by parallel computations. Since all HGon topological parameters are independently controlled, structures can be generated and analyzed in parallel, limited only by available computational resources. Work is also in progress to increase FE computation speeds using newly available GPU-based finite element codes (e.g. NiftySim^27^).

In Part 2 von Mises stress was used for meta-structure optimization, which seemed an obvious first choice—but there are many other possibilities. Optimization using *combined fields* are primary ongoing research. Novel objective functions and mappings are being investigated; including strain energy, shear stress, modal stress, electromagnetic, heat transfer, fluid flow and many other potential fields could lead to unprecedented designs and performance of HGon and related meta-structures.

An issue with any type of lattice or meta-structure is discretization; i.e. resolving fine surface features. Consolidation or ‘loss’ of fine features is inevitable when surface constraints are relaxed. In practice this can actually be an *advantage* for overall design of the component. For example, in the process of design there are often issues that might be better addressed with a lower density conforming HGon meta-structure. Consider the question: is the ‘lost’ edge as shown in Fig. 11*critical* to the overall design? If so, then the higher density HGons are preferred. If not, then the lower density HGon (Fig. 11A) is likely better from a strength-to-weight ratio perspective. Addressing these and other heuristic parameters using Machine Learning and Artificial Intelligence methods are an important focus of future work for next-generation HGon meta-structures.

The damping method used here (viscoelastic dissipation) is certainly a gross simplification of ‘true’ frictional or thermal-related damping energy loss. However, the same material model was used *identically* for all lattices and equivalent HGon meta-structures, therefore the comparisons reported here (e.g. total stresses, end vibrations, and damping) are due to differences in topology, not the material model. More realistic models of damping and fatigue are in progress. Fractional-order models^28^ are of particular interest because of the potential for relating *fractional* material parameters to HGon meta-structure parameters.

## Summary and conclusion

This study presented and analyzed a new type of meta-structure that can enable unprecedented vibration control, conform to complex shapes, and greatly reduce mass/weight for both simple bricks and complex, multi-connected structures. Results from both Part 1 and Part 2 demonstrated that HGon structures had (surprisingly) near total elimination of higher frequency vibrations as compared to the original CAD solid/shell and periodic lattices. High stress concentrations that appeared in the lattices were also largely reduced or even eliminated in the HGon meta-structures, indicating that HGons can improve overall component fracture and fatigue strength. These methods and resulting data are valuable for guiding future research in both computational and experimental designs for real-world testing and comparison with equivalent FEA analyses. The HGon generation/analyses methods presented here can be applied to any component where ultra lightweight, ultra strength-to-weight ratios, and vibration control are a priority—such as in aerospace, automotive, bioengineering, architecture, footwear, military-ballistics, sportswear, protective gear, and many other categories. Results also provide valuable initial baseline data for future research using Machine Learning and Artificial Intelligence methods to investigate novel designs for complex, multi-material, next generation ‘smart’ meta-structures.

Lattices and trusses have been used effectively for centuries in the design of bridges, buildings, towers, and a great many other applications. This study presented a new type of meta-structure that can, to our knowledge for the first time, enable fully conforming, optimized lattice-type and hybrid meta-structures. HGon meta-structures are a first step towards a new class of innovative ultra-light and strong meta-structures with new strategies for meta-optimization of high performance components.

## Supplementary Information

Supplementary Information 1.

Supplementary Video 1.

Supplementary Information 2.

Supplementary Information 3.

Supplementary Information 4.
