# Supplementary material for: FE vibration analyses of novel conforming meta-structures and standard lattices for simple bricks and a topology-optimized aerodynamic bracket
Source: Sci Rep. 2020 Dec 8;10:21484. doi: 10.1038/s41598-020-78239-9 (PMC7722937; doi:10.1038/s41598-020-78239-9)
Supplement: Supplementary file 1 — Supplementary Information 1. [file 41598_2020_78239_MOESM1_ESM.docx]

**APPENDIX**

Manuscript titled: “FE vibration analyses of novel conforming meta-structures and standard lattices for simple cuboids and a topology-optimized aerodynamic bracket”

Todd Doehring, William Nelson, Thomas Harris, Alan Freed

**1. Methods and Results, videos and data:**

- Included in supplementary materials is a full summary of this study (3 min). A higher resolution version is provided here for convenience: [https://youtu.be/V_I81DYqH24](https://youtu.be/V_I81DYqH24k) (skip to time=45 sec for example 3D vibrations).

- A zip file containing raw vibration end-displacement and stress data is provided in supplementary materials. [Link to zip archive](https://drive.google.com/file/d/181nyIX4byYp4HR6VMEeSxVmiDmipb_bG/view?usp=sharing) (for convenience). Note that data is in LibreOffice spreadsheet (.ods) format.

**2. Additional videos describing HGon technologies:**

- Basics of HGons: computing L1-2 Meta-structures (very short, 1 min): [Youtube link](https://youtu.be/unLOuyc9pa8).

- New HGon technologies (2.5 min). Examples of exotic structures for vibration and ultralight components: [Youtube link](https://youtu.be/a7L0LXZ9bdo).

- Recent developments in our field-structure optimization methods: [Youtube link.](https://youtu.be/uLXZJzElDhM)

- Image gallery of various HGon structures: <https://www.abemis.com/galleries.html>

**3. 3D model data:**

- Selected 3D models: STL files can be provided upon request. Many HGon models can be found here including the Aerodynamic bracket: <https://sketchfab.com/tcdoeh/models>

Note: due to proprietary information and NDA issues, all models can be viewed but only selected models are ‘downloadable”.

- Test shape ‘teardrop’ surface model (see below for further description) in STL format (supplementary material).

- Base HGon generated from the teardrop Test Shape in STL format (supplementary material)

**4. Description and data for an additional example Test Shape:**

We have developed several test shapes (one shown below) that we use to evaluate our HGon (or any lattice-type) meta-structures. This challenging teardrop shape has multiple complex features such as adjoining flat and highly curved surfaces, sharp edges, strong taper and tip, and multi-connected overlapping oval and rectangular holes.

This teardrop test-shape geometry and a base HGon meta-structure (nominal 2mm strut diameter) are provided in the supplementary material in STL format. Readers can thus apply their own methods and compare. We hope this test-sample is useful for comparing methods.

Contact the Authors for questions or any additional information on this or other shapes.


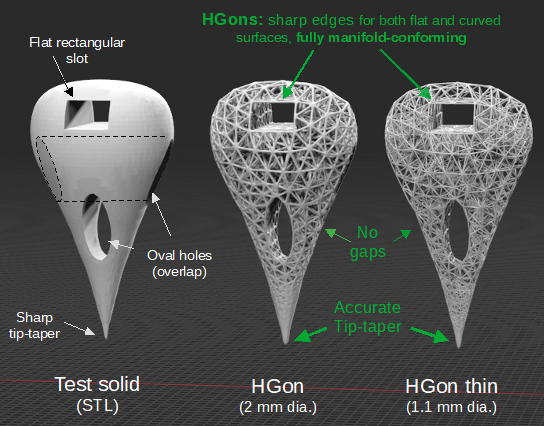
Figure A-1. Shown above is one of the test solids (left) that we use to evaluate our HGon generation methods and results. Two HGon structures with different diameters (right) are also shown. This structure has a number of challenging features. Files for these structures (STL format) are provided in the Supplementary Materials.

**5. Description of vector-sum versus single direction oscillation:**

It might not be immediately obvious the difference between the vector-sum (i.e. total) displacement vs. time used in this study versus a single direction displacement (e.g. X, Y, or Z direction). Below are plots of the top displacement oscillations of a simple rectangular shell (as in Fig. 14-A) to illustrate.

Figure A-2. Plots of top displacement (mm) vs. time (s) for a shell structure showing X-direction (gray) and vector-sum total (green) displacement oscillations post-release.

The X-direction displacement (in the loading direction) shows approximately symmetric oscillation while the vector-sum (total) displacement has only positive values. The vector-sum is a more succinct and sensitive measure of differences because post-release vibrations of this 3D structure have smaller but still significant Y and Z components. Due to the vector sum calculation vsum = sqrt( x^2 + y^2 + z^2), the apparent period of vector-sum oscillation is 2x the actual component oscillation period.
